# Narrative visualizations: Depicting accumulating risks and increasing trust in data

**DOI:** 10.1186/s41235-025-00613-w

**Published:** 2025-02-21

**Authors:** Madison Fansher, Logan Walls, Chenxu Hao, Hari Subramonyam, Aysecan Boduroglu, Priti Shah, Jessica K. Witt

**Affiliations:** 1 Department of Psychology, University of Michigan, Ann Arbor, USA; 2 Department of Intelligent Systems, Delft University of Technology, Delft, The Netherlands; 3 Graduate School of Education, Stanford University, Stanford, USA; 4 Department of Psychology, Koç University, Istanbul, Turkey; 5 Colorado State University, Fort Collins, USA; 6 Department of Physical Medicine & Rehabilitation, University of Michigan, Ann Arbor, USA

**Keywords:** Data visualization, Misinformation, Risk perception

## Abstract

In contexts where people lack prior knowledge and risk awareness—such as the COVID-19 pandemic—even truthful visualizations of data can seem surprising. This can lead people to mistrust the veracity of the data and to discount it, leading to poor risk decisions. In this work, we illustrate how narrative visualizations can achieve a balance between the benefits of three common risk communication mediums (static visualizations, interactive simulations, and affect-laden anecdotes). We demonstrate empirically that viewing a narrative visualization mitigates the reduced concern induced by a static visualization when communicating COVID-19 transmission risk (Study 1). Through mediation analysis, we show that narrative visualizations are more effective than static visualizations at increasing concern about large risks because they increase one’s perceived understanding and trust in data (Study 2). We argue that narrative visualizations deserve attention as a distinct class of visualizations that have the potential to be powerful tools for scientific communication (especially in contexts where data are surprising, and empiricism is important).

**Supplementary Information:**

The online version contains supplementary material available at 10.1186/s41235-025-00613-w.

## Introduction

During our lifetimes we repeatedly expose ourselves to extremely small risks. For example, the risk of experiencing skin cancer increases with exposure. to UV radiation. To date, there has been a dearth of research on how to best communicate small but accumulating risks, such as the risks associated with the continuous exposure to small amounts of foreign/dangerous substances (e.g., heavy metal poisoning) or the repetition of risky behaviors (e.g., driving without a seat belt; Slovic,et al., 1978). Prior research suggests that cumulative risks tend to be underestimated on average (Doyle, 1997; Slovic, 2000), though this average may be based on a significant number of individuals who severely underestimate risk and another who slightly overestimate risk (De La Maza et al., 2019). These types of risks are harder to communicate not only because humans are typically bad at accumulation-based judgments, but also because the overall accumulated risk is perceived to be somewhat implausible, leading to a mistrust of data. Thus, communicative efforts need to focus not only on identifying the most appropriate format to depict the accumulation pattern, also on finding ways to increase people's trust in the end result.

People commonly use charts or other static visualizations to communicate information about complex systems like climate change and global pandemics (Franconeri et al., 2021). For instance, a visualization designer might use an icon array that illustrates a rise in temperature over time to highlight the effect of human activity on climate. Such visualizations effectively convey specific data or outcomes (e.g., that global temperatures have risen alongside carbon emissions), but they do not illustrate the underlying mechanisms that generate them (e.g., the greenhouse effect). As a result, viewers may not understand the visualization’s intended message (Newell et al., 2016) and may even discredit the data if it seems surprising or inconsistent with their prior beliefs (Lord et al., 1979; Rhodes et al., 2014; Shah et al., 2017). From an individual’s perspective, mistrust in data could be related to various personal factors such as one’s political identity (Peck et al., 2019) and one’s lack of understanding of how the data arise—especially in data showing complex systems such as the exponential growth of disease prevalence (Fansher et al., 2022a; Witt et al., 2022) or visualizations illustrating uncertainty (Padilla et al., 2022a). Furthermore, one’s misunderstanding of the purpose of science models (e.g., believing that these models should make accurate predictions or depict reality) may also lead to distrust in data (Witt et al., 2022). In the current study, we examined methods for communicating cumulative risk: namely with narrative visualizations, static visualizations, and anecdotes. We also examined whether one’s trust and understanding of the data mediated the relationship between the presence of a visualization and change in behavior or risk understanding.

### The role of anecdotes

One approach for risk communicators is to avoid discussing the data altogether, and instead give an anecdote about an individual that was negatively affected by the risk. This approach is often used to increase concern about low-risk events. In fact, prior research has shown that anecdotes may be more persuasive than providing statistical data in health-related contexts, especially when personal decisions are involved or when threats are serious (for a review/meta-analysis, see Freling et al., 2020). Anecdotes are thought to be effective because they are concrete and easier to understand than statistical evidence, are emotionally interesting, and, possibly, more memorable (Freling et al., 2020). For example, successful texting and driving interventions often rely on stories about texting and driving accidents rather than presenting data about accident risk (Cutello et al., 2020). In general, stories that tug at one’s emotions, increase fear, and provide visual imagery tend to be persuasive (Cutello et al., 2020).

Unfortunately, communicating risks solely with anecdotes is potentially problematic and may hinder critical thinking. For example, Rodriguez et al. (2016) found that people were less likely to notice flawed conclusions when anecdotes were presented in descriptions of science studies in the media. Furthermore, people often become inured to anecdotes after multiple exposures (Slovic et al., 2017). Importantly, anecdotes are also not effective in all situations (Zebregs et al., 2015). A meta-analysis by Zebregs et al. (2015) found that statistical data were generally more influential on attitudes and beliefs than anecdotes, while anecdotes were more effective at changing behavioral intentions. For example, anecdotes were less likely to alter readers’ attitudes toward climate change, seat belt use, or exercise than statistical data. In contrast, anecdotes had a larger influence than statistical data on behavioral intentions with respect to risky sexual behavior, exercising, and tanning bed use. A more recent and somewhat broader meta-analysis suggests that anecdotes can also be more persuasive than statistical data when the risk consequences are high, and when they are related to personal decisions rather than decisions about others. Interestingly, one study found that individuals reported preferring statistical data to anecdotes for making decisions (Freling et al., 2020). Thus, although anecdotes may be able to increase risk concerns (at least when risks are severe and personal), they are not a panacea for communicating risks to the public. These drawbacks call for a method to convey data effectively without relying on anecdotes. One possibility is the use of data visualizations depicting risk.

### Visualizing risks

#### Static visualizations

One approach to help individuals understand risk and frequency data, especially individuals with low numeracy, is to present icon arrays or similar visualizations (Galesic & Garcia-Retamero, 2011). Often, pairs of icon arrays are used to present relative and absolute risks. Icon arrays are especially helpful for communicating just how small risks might be. For example, a recent study showed that presenting a very small risk (i.e., the risk of serious side effects from the COVID-19 vaccine) as an icon array (one in 1 million dots) was effective in communicating the rarity of a ~ 0.000001% risk (Fansher et al., 2022b). This representation led to much lower concerns about COVID-19 vaccines and increased positive attitudes toward vaccination.

While prior work demonstrates that visualizations like icon arrays are effective at communicating risks, icon arrays are still static visualizations, meaning that they provide no mechanistic explanation for how the larger-than-expected (or smaller-than-expected) risk calculations are derived—they merely present the final risk estimate. Therefore, they may not be as compelling to the viewer, and may be less likely to increase trust in data when compared to visualizations that include information about how the risk estimate was derived.

#### Interactive simulations

One way to provide people with a richer understanding of data is to provide them with interactive simulations that demonstrate how data change under different scenarios. Interactive simulations change how people view data, especially data about different potential future outcomes. Rather than thinking of outcomes as fixed, they recognize that science models are tools for examining the consequences of different changes (Herring et al., 2017; Sterman, 2011). For example, Herring et al. (2017) gave participants simulations that showed how different emissions scenarios led to different magnitudes of climate change. They found that using those simulations increased people’s concerns about climate change. Similarly, Witt et al. (2022) showed that interacting with simulations explaining exponential growth of disease spread as well as the impact of various public health policies increased people’s understanding and trust in social distancing recommendations.

Although interactive simulations are often effective, they have several potential limitations. First, interactive simulations require more time and resources to create than static simulations. Second, individuals must be motivated to interact with simulations and manipulate relevant variables. If too little or too much guidance is provided for exploring a simulation, individuals may simply not engage (Adams et al., 2015). Third, individuals can engage in random or mindless interactions rather than systematically exploring the effects of different variables (Liew et al., 2014) and such visualizations may contain elements that are distracting (Hegarty, 2004; Mayer, 2005). Finally, simulations require individuals to understand the mapping between different visualizations, which is often challenging (Magana & Silva Coutinho, 2017).

#### Narrative visualizations

The two types of visualizations mentioned above fall at the extreme ends of a spectrum. Static visualizations communicate efficiently but offer little mechanistic explanation, while simulations offer detailed mechanistic information but require more of the viewer’s time and the designer’s careful guidance. In contrast, narrative visualizations (Segel & Heer, 2010) strike a balance between these two extremes. These visualizations use step-by-step explanations to convey details (like simulations) but constrain the viewer’s inquiry to a predefined set of insights (like static visualizations). Visualizations that meet this definition already appear in popular publications such as the New York Times (Buckley et al., 2022; Byrd et al., 2022) and Reuters (Cage, 2021; Dutta et al., 2019; Levine et al., 2021), but to our knowledge they have not been identified as a specific group of visualizations or studied specifically in the context of risk communication. Okan et al. (2015) examined different factors that may improve the efficacy of icon arrays for communicating risk. They found evidence that showing data in the form of icon arrays alongside explanatory labels increased risk understanding, particularly in participants with low graph literacy, suggesting that narrative visualizations may be an effective tool for presenting risk information.

Narrative visualizations have been widely used by visualization designers to ensure readers attend to key data related facts (Bach et al., 2018; Hullman & Diakopoulos, 2011; Hullman et al., 2013; Segel & Heer, 2010). Typically, these visualizations incorporate external guidance such as sequential panels (e.g., the martini glass approach) and annotations to draw readers’ attention through the data in the appropriate order. In these approaches, the goal is to control how readers comprehend the visualization by ensuring coverage of key facts and associated narrative context, but not necessarily to increase understanding about the data-generating process (i.e., how the facts occurred). For instance, Lee et al. (2021) highlighted the ways in which coronavirus skeptics effectively used counter-visualizations to promote their anti-mask stances. Reinholtz et al. (2021) showed that participants presented with inflow visualizations (new coronavirus cases each day) judged coronavirus risk as lower compared to participants presented with stock visualizations (cumulative number of coronavirus cases). The subset of narrative visualizations that focus on explaining the data-generating process might be especially appealing for complex and empirically driven communication challenges like cumulative risk.

### Trust and data visualization

The literature on trust in data visualization has proliferated in recent years, likely due to a need for improved scientific communication in an era where misinformation threatens public health (Cook et al., 2015; Roozenbeek et al., 2020) and people are increasingly skeptical toward science (Rutjens et al., 2018). While data visualizations have been shown to be effective at communicating risk information (Lipkus & Hollands, 1999), especially in the context of health risks (Garcia-Retamero et al., 2012), researchers suggest that *trust* in the presented data is necessary in order to update their beliefs and behaviors accordingly (Garcia-Retamero & Cokely, 2017; Mayr et al., 2019). Researchers have examined how the features of visualizations influence trust in various contexts, such as viewing election forecast visualizations (Yang et al., 2023), and the role of visualization features such as processing fluency (Elhamdadi et al., 2022a), title alignment (Kong et al., 2019), and the communication of uncertainty (Gustafson & Rice, 2020; Hullman, 2019; Kerr et al., 2023; van der Bles et al. (2020); Yang et al., 2023).

There are many challenges with measuring and defining trust in the context of data visualization (Elhamdadi et al., 2022b, 2023). In the current study, we adopt the general definition of trust in visualization from Mayr et al, (2019, p. 25), in that “trust is the user’s implicit or explicit tendency to rely on a visualization and to build on the information displayed.” Since the time of this study, other authors have presented multidimensional frameworks describing the various facets of trust in visualization (e.g., Elhamdadi et al., 2023; Pandey et al., 2023). For example, Pandey et al. (2023) described trust in visualization as being based on data credibility, clarity, reliability, familiarity, and confidence. Trust in visualization is also commonly referred to as a type of interpersonal trust between the trusted (visualization) and trustee (user) (Kelton et al., 2008; Lewicki et al., 2006; Zhao, 2017). Wang et al. (2014) described trustworthiness as referring to the honesty and believability of the source, with people being more likely to adopt suggestions from trustworthy sources and be influenced toward positive attitude change.

Indeed, in Garcia-Retamero and Cokely’s (2017) review on the effect of data visualization on risk literature, the authors state that “Well-designed visual aids robustly improve risk understanding by encouraging more thorough deliberation, facilitating self-assessment, and reducing biased risk representations, which in turn benefit attitudes, behavioral intentions, and trust, leading to healthier decisions and more positive health outcomes” (p. 622). There is a plethora of research suggesting that visual aids can lead to improved risk understanding (e.g., Garcia-Retamero et al., 2012, Garcia-Retamero & Cokely, 2017; Lipkus & Hollands, 1999; Zipkin et al., 2014), especially for individuals with low numeracy (Garcia-Retamero & Cokely, 2014). ome researchers suggest that data visualization can also lead to increased trust in data. Petrova et al. (2015) found that viewing visualizations of prostate cancer risk was associated with increased risk comprehension which was in turn associated with a willingness to engage in shared decision-making with a physician, suggesting that viewing the risk visualization increased trust in the provider.

### The current study

Anecdotes, static visualizations, interactive simulations, and narrative visualizations are all used to communicate data about risk in popular press, but each contain different types and amounts of information, as discussed above. In the current study, we examine  the potential benefits of narrative visualizations for communicating cumulative risk. Prior research on visual communication of risk offers guidance on increasing concerns about underestimated risks, but it does not address the communication of cumulative risk. Rather, much of the prior work focuses on helping individuals comprehend magnitudes of individual risks. For example, in public health contexts perceived risk is a necessary precondition for people to change their behavior (van der Pligt, 1996).

In this work, we focus on narrative visualizations because they convey information about data lineage—which is typically not conveyed by some other commonly used mediums like static visualizations and anecdotes. We argue that, because they communicate information about the data-generating process, narrative visualizations could be powerful tools for scientific communication - particularly in risk contexts where people may be harmed if they make decisions based on inaccurate or incomplete information. We used the COVID-19 pandemic to empirically evaluate the effectiveness of narrative visualizations for communicating accumulating, quantitative risk information.

Study 1 compared the effectiveness of narrative visualizations with other common risk visualizations for communicating the cumulative risk of COVID infection at a Thanksgiving dinner. The influence of communicated data on people’s attitudes and decision-making can also depend on people’s *trust* in data, calling for more focused interdisciplinary research on the relationship between trust and data visualization (Borgo & Edwards, 2020; Mayr et al., 2019; Park & Gil-Garcia, 2022). In a follow-up study, we investigated the mechanism behind the narrative visualization’s effectiveness and found that narrative visualizations increased trust in the data by increasing feelings of understanding, while anecdotes did not significantly affect understanding.

## Study 1

The goal of Study 1 was to use the context of the COVID-19 pandemic to test the effectiveness of narrative visualizations for communicating accumulating, quantitative risk information. In November 2020, just a few days prior to the USA Thanksgiving Holiday, we tested how different data presentation methods (i.e., anecdotes, static visualizations, and narrative visualizations), differing in their amount of information about the data-generating process and appeal to emotion, would differentially impact people’s COVID-19-related plans and concerns during the Thanksgiving season. The materials for all the conditions were designed to closely mimic COVID visualizations found in the popular press, while prioritizing legibility of the text and graphs. We tested participants immediately before Thanksgiving and in a follow-up study a few weeks after to confirm whether the presentation in fact led to any changes in plans. The complete materials, code, and data are available at https://osf.io/k43ev/?view_only=67cc6401b06946889ac499fc19afec2f. For exact wording of all the survey items, please see the Supplementary Materials.

### Methods

#### Participants

Complete data were collected from 1592 US participants (770 female; age M(SD) = 33.54(11.96)) recruited from Prolific on Nov 25, 2020, a day before Thanksgiving, to complete session 1. Participants received an average pay of $10.30/hr (USD), and the median completion time for the study was 4 min and 40 s. A total of 1276 participants (622 female; age M(SD) = 34.02(12.10) years) returned for the second part of this study (session 2) conducted from December 22, 2020 to January 1, 2021. Participants received an average pay of $9.29/hr (USD), and the median completion time for the study was 5 min and 10 s. All procedures were determined to be exempt by the University of Michigan IRB.

#### Design

Participants were randomly assigned to one of four conditions where they viewed either a static visualization (Static Condition, *n* = 398), the same static visualization combined with an anecdote (Static + Anecdote Condition, *n* = 399), an anecdote presented without a visualization (Anecdote Condition, *n* = 398), or a narrative visualization (Narrative Visualization Condition, *n* = 397).

#### Materials

*Pre-Intervention Materials*. Participants were asked to indicate who they planned to spend Thanksgiving with (alone/with household, extended family, friends/neighbors, strangers). To measure concerns about COVID-19, participants responded to two questions:

1. How concerned are you about getting COVID-19/Coronavirus at Thanksgiving?
2. How concerned are you that someone in your family will get COVID-19 at Thanksgiving?

Participants rated their level of concern for each of these questions on a scale of 0 (*not concerned at all*) to 100 (*extremely concerned*) using slider scales (anchored at 50). To measure participants’ perceived risk of dining with an infected individual at Thanksgiving dinner, we asked: “What is the risk that at least one person at a Thanksgiving table with 10 people has COVID-19?”, which they responded to on a scale of 0–100% with a slider scale.

##### Intervention materials

**Fig. 1:**
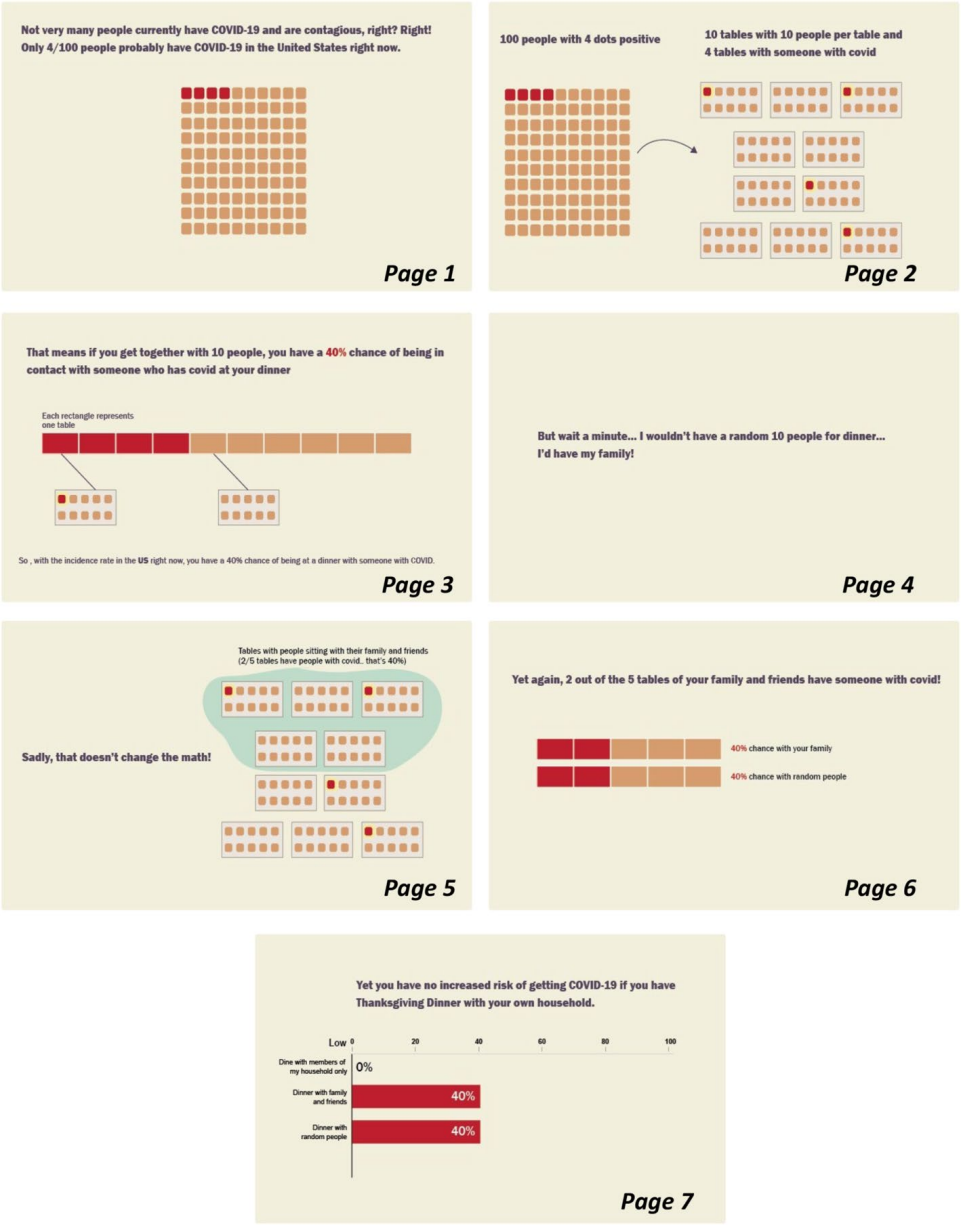
Narrative visualization intervention materials for Study 1. The page numbers were added to the figure to show the order in which the materials were presented to participants

*Narrative Visualization Condition.* Our narrative visualization intervention presented participants with a series of icon arrays illustrating the spread of COVID in a fictional community during Thanksgiving. The narrative visualization provided a step-by-step explanation for how there is a 40% risk of COVID transmission at a Thanksgiving dinner of 10 people if there was a 4% infection rate in the fictional community (Fig. 1). The narrative visualization materials were specifically inspired by the FiveThirtyEight article, “Why Even A Small Thanksgiving Is Dangerous” (Koerth & Elena, 2020).

*Static Visualization Condition.* The Static Visualization Condition served as a baseline to compare with the other conditions. This condition was not intended to evoke an emotional reaction like an anecdote and did not provide information about how the data were calculated like the narrative visualization. Participants in this condition viewed the last page of the narrative visualization (see Fig. 1, page 7)—a bar graph showing that there would is a 40% risk of transmission at a Thanksgiving dinner with 10 people in this scenario. Participants were not provided with a step-by-step explanation about how that number was calculated.

**Fig. 2:**
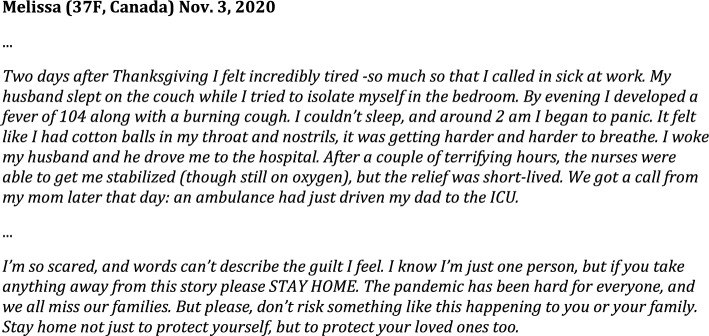
Anecdote intervention materials. *Note.* Participants read an anecdote from someone who had contracted COVID at Canadian Thanksgiving. The below is a representative excerpt from the anecdote. The full text is one page in length and is available in the Supplementary Materials

*Anecdote Condition.* Participants in the Anecdote Condition read an anecdote describing an individual’s personal experience with COVID-19 after contracting it at Canadian Thanksgiving (which occurs prior to USA Thanksgiving). The anecdote was written by conducting an informal survey of social media posts describing people’s experiences with COVID-19. The details from multiple posts were merged with the goal of creating a cohesive story to describe a serious case of COVID-19, evoking an emotional response (see Fig. 2 for the anecdote).

**Fig. 3:**
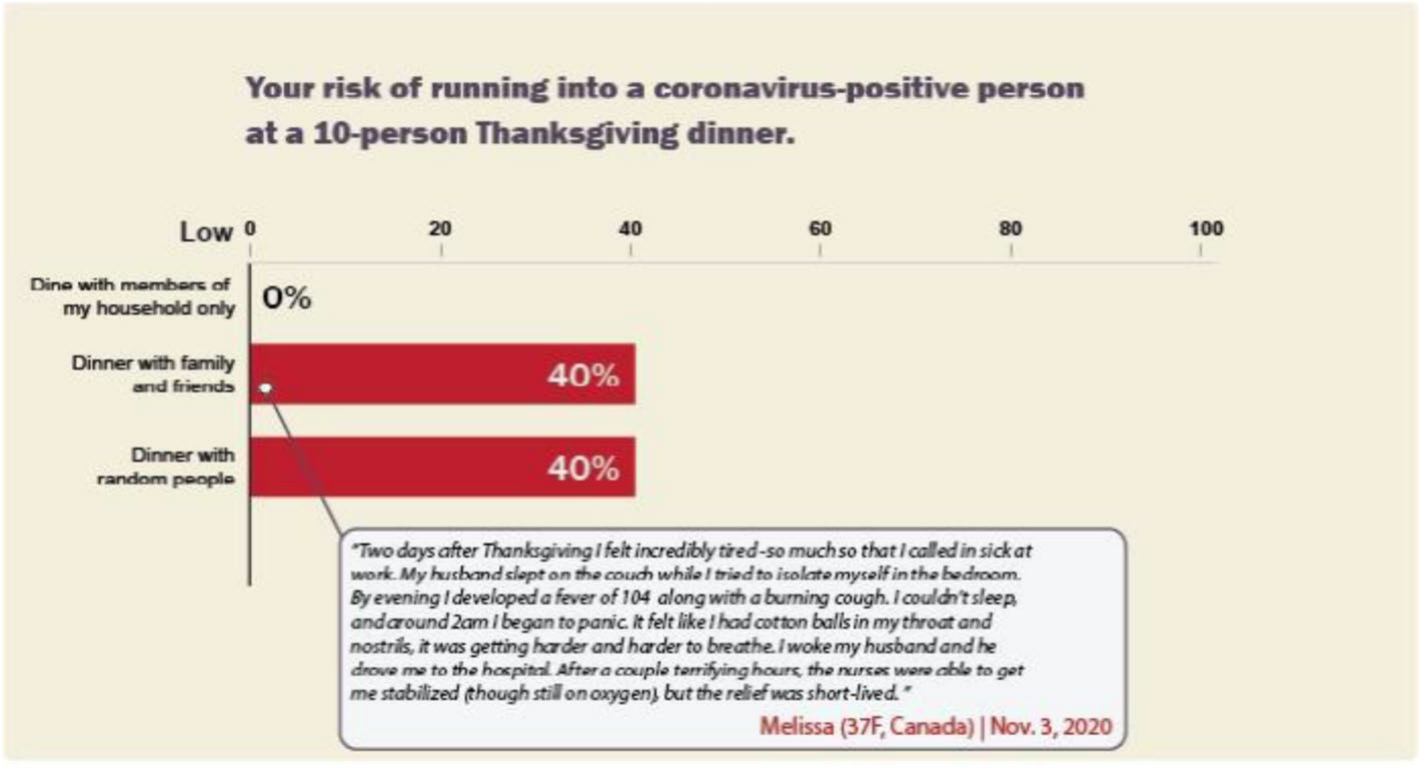
Static + Anecdote Condition materials for Study 1. *Note.* Participants in the Static Condition viewed the bar graph without the anecdote

**Fig. 4:**
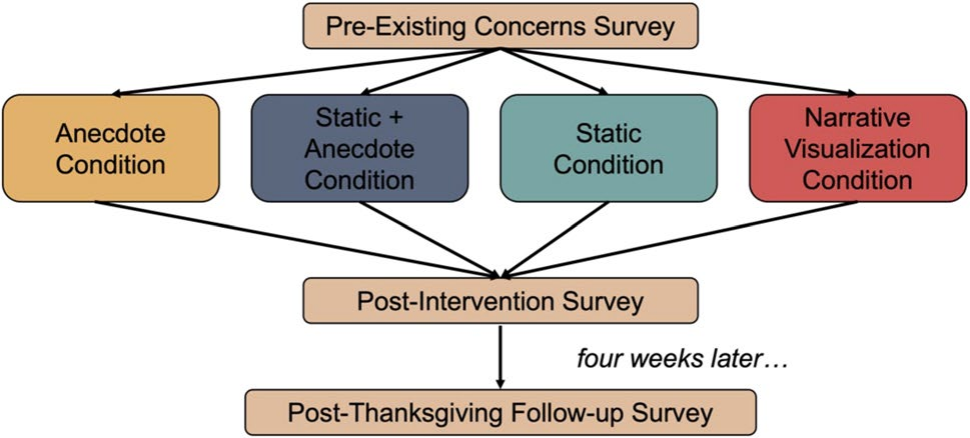
Study 1 task flow

*Static* + *Anecdote Condition.* The Static + Anecdote Condition (see Fig. 3) presented the same graph as the static intervention, but included a text box containing one representative paragraph from the Anecdote Condition’s materials (Fig. 2). We included the Anecdote and Static + Anecdote Conditions to see how narrative visualizations compared to typically successful interventions in the literature on medical risk assessment (i.e., using emotional-laden content, rather than explaining the data or showing data without information on how they were generated) (Fig. 4).

*Post-Intervention Survey*. After viewing the intervention materials, participants completed a post-intervention survey which  again assessed their attitudes and concerns regarding COVID-19. They answered the same two concern items and the perceived risk item as in the pre-intervention survey. They were also asked the question: “How do you feel about your current plans?” to which they responded with a slider scale from 0 (*not concerned at all*) to 100 (*extremely concerned*). While change in concern, change in current plans, and change in perceived risk are the main variables of interest in our analysis of the Session 1 data, participants also answered demographic questions, and disclosed their plans for the December/January holidays. All items are available in the Supplementary Materials.

*Post-Thanksgiving Follow-up Survey*. To assess the longevity of any changes in concern, and to determine whether the narrative visualization affected participants’ actual behavior (i.e., holiday plans), we followed up with participants after Thanksgiving. For all items, please see the Supplementary Materials.

Participants were first asked whether they planned to attend or host/have attended or hosted any in-person gatherings with people outside of their household for the December/January holidays (Yes, No, Unsure). We also asked whether COVID had changed their holiday plans (No change; spending the holidays with fewer people than planned; spending the holidays with more people than planned). We again assessed perceived risk of COVID with the item: “Based on the previous Survey of Holiday Plans, what is the risk that at least one person at a Thanksgiving table with 10 people might have COVID-19?” to which participants responded with a slider scale from 0 to 100%.

To see whether intervention condition influenced participants’ Thanksgiving activities, we asked them “Did you make any last-minute changes to your Thanksgiving plans?” (yes/no) followed by “Did your change of plans increase or decrease the number of people you interacted with during Thanksgiving?” (increased, decreased, neither increased nor decreased). We also assessed COVID concern with two items:

1. How concerned are you about getting COVID-19/Coronavirus via social gatherings in December? (slider scale of 0 (*not concerned at all*) to 100 (*extremely concerned*))
2. How concerned are you that someone in your family will get COVID-19 via social gatherings in December? (slider scale of 0 (*not concerned at all*) to 100 (*extremely concerned*))

#### Procedure

Prolific workers were invited to participate in our initial survey, conducted before American Thanksgiving on November 25, 2020. After providing informed consent, participants completed the pre-intervention survey questions and were randomly assigned to view the materials associated with one of the four experimental conditions (Static, Static + Anecdote, Narrative Visualization, or Anecdote). They then completed the post-intervention survey questions followed by Subjective Numeracy Scale (SNS-3, McNaughton et al., 2015) and demographic questions. A few weeks later, participants from the first part of the study were invited to complete the Post-Thanksgiving survey. This survey was available to participants from December 22, 2020 to January 1, 2021. Participants again provided informed consent and answered the questions described above. The exact wording and ordering of all the questions from Study 1 are available in the Supplementary Materials.

### Results

The results presented are guided by our main questions of interest:

1. Are narrative visualizations more effective than static graphs at increasing concern and perceived risk?
2. How effective are narrative visualizations relative to anecdotes?
3. Does adding data to the anecdote condition (Static + Anecdote Condition) make the intervention more effective than just viewing one or the other?

Given that we present the results of 20 various tests, we used a Bonferroni correction to reduce the likelihood of Type I error. Thus, in the following analysis, we decreased our significance level to *p* < 0.003 ( $\alpha =\frac{.05}{20}= 0.0025$).

#### Data exclusion

Our analysis focused on participants who indicated before intervention that they planned to have a meal with people outside of their home (*n* = 532), as these were the participants who were not taking proper precautions. It can be assumed that that they perceived a lower risk of contracting COVID-19 than was appropriate. Please see Table 1 for the demographic characteristics and sample sizes for the participants from each session after applying our exclusion criteria.

**Table 1:** Sample sizes and demographic features of samples included in the analysis

	Anecdote Condition	Static + Anecdote Condition	Static Condition	Narrative Visualization Condition	Total N	Age M(SD)	Gender
Pre-Thanksgiving (Session 1)	135	150	131	116	532	33.91(11.24)	242 F
Post-Thanksgiving (Session 2)	104	108	104	91	407	34.02(11.33)	201 F

#### Pre-thanksgiving data analysis

*Change in Concern*. We calculated pre- and post-intervention concern scores for each participant by averaging their responses to the two concern questions in each of the pre- and post-intervention surveys (i.e., How concerned are you about getting COVID at Thanksgiving? How concerned are you that someone in your family will get COVID at Thanksgiving?). Note that the responses analyzed here were only from people who planned to gather with other people for the 2020 Thanksgiving holiday, suggesting that their concern was lower than recommended by public health authorities at the time. Therefore, we treat an increase in concern as a positive outcome in this study. That said, it is worth noting that an increase in concern is not always a desirable outcome. More generally, we hope that participants understood the information presented and updated their beliefs based on that information.

**Fig. 5:**
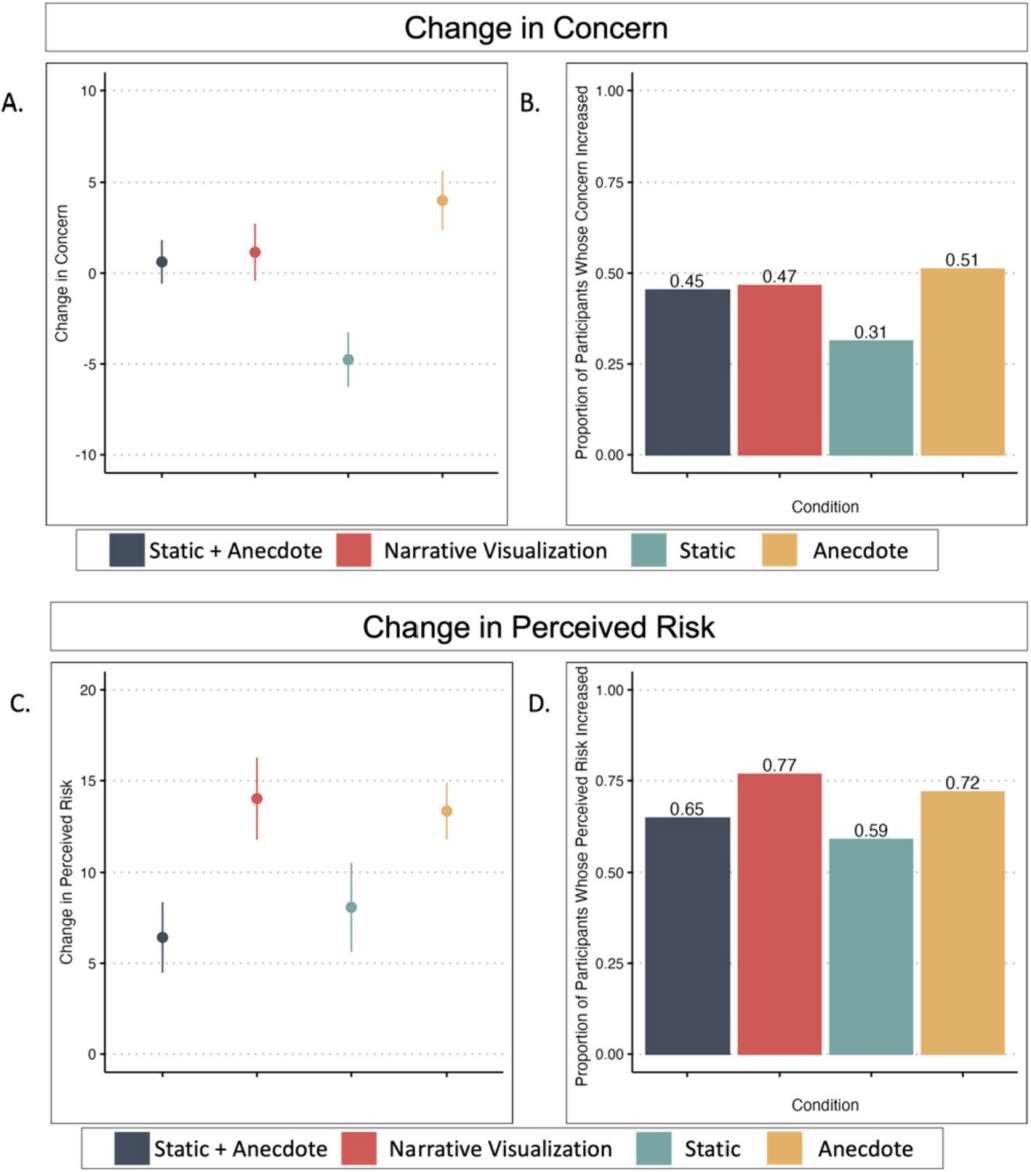
Study 1 Change in Concern and Perceived Risk by Condition. *Note.* Figure 5a illustrates mean and standard error change in concern by condition ($postconcern-preconcern$). A change in of zero indicates no change in concern; positive scores indicate increased concern, and negative scores indicate decreased concern. Figure 5c illustrates mean and standard error change in perceived risk ($postrisk-prerisk$). Figures 5a and 5c are not on the same scale. Figures 5b and 5d illustrate the proportion of participants whose concern or perceived risk *increased* after viewing their respective intervention

**Fig. 6:**
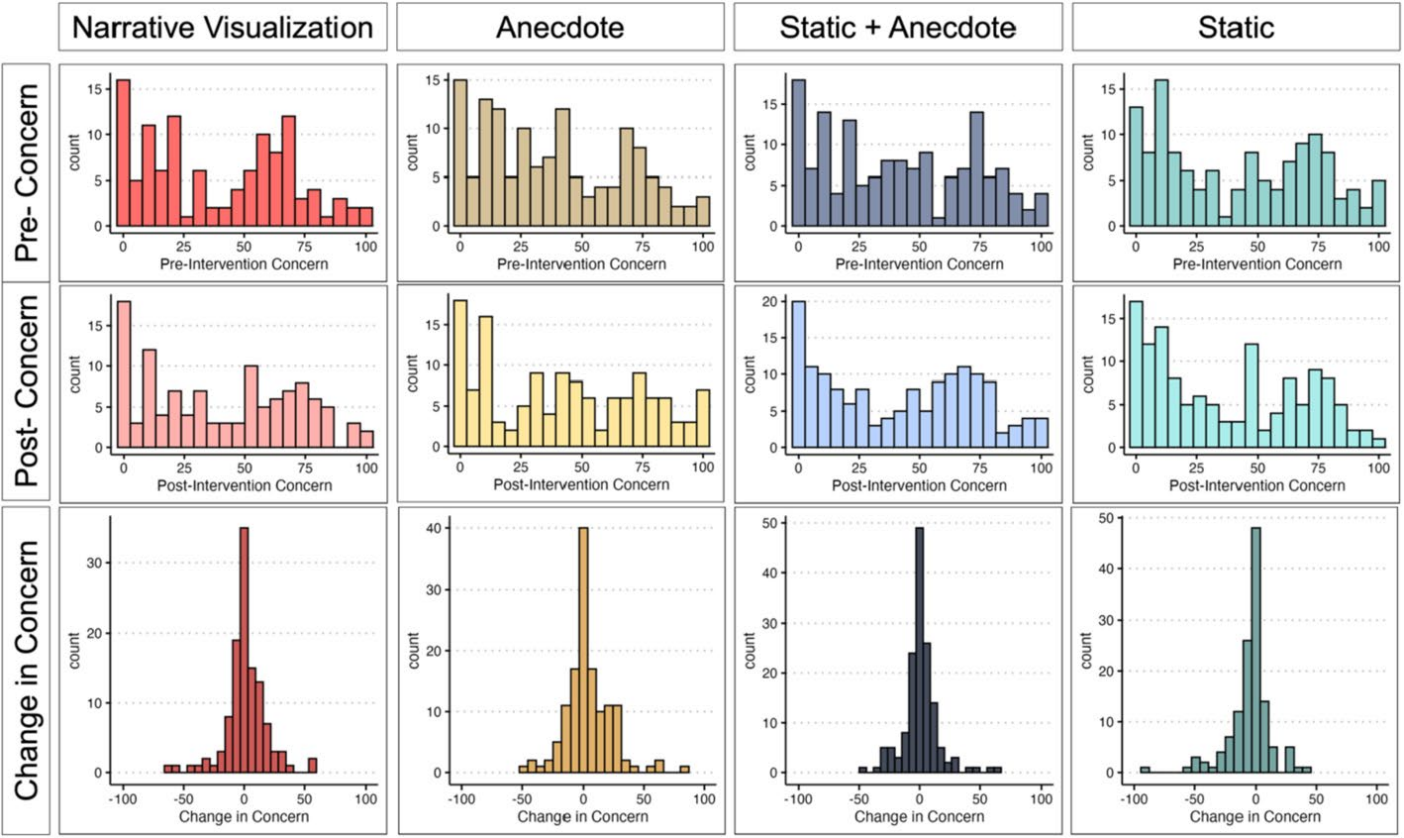
Distributions of Concern Scores

To compare post-intervention concern across intervention conditions, we ran an ANCOVA with condition as the predictor and pre-intervention concern as a covariate.The Static Condition served as the reference group for the condition comparisons (see Fig. 5a and Table 2). We found evidence that the Static Condition reported lower concern post-intervention than the Anecdote Condition (*p* < 0.001), and marginal evidence that the Static Condition reported lower concern post-intervention than both the Static + Anecdote (*p* = 0.008) and Narrative Visualization (*p* = 0.009) Conditions. The distributions of pre-intervention concern, post-intervention concern, and change in concerns are available in Fig. 6. We would like to note that many of the participants did not update their concern score from pre- to post-intervention (see *Exploratory Analysis* for more detail).

**Table 2:** Change in Concern and Perceived Risk from Pre- to Post-Intervention in Session 1

ANCOVA Results—Session 1 (Post-Concern ~ Condition + Pre-Concern)			
Coefficients	Estimate	*t* value	*p*
Intercept	0.73	0.41	0.68
Static | Static + Anecdote	5.26	2.67	0.008
Static | Narrative Visualization	5.50	2.61	0.009
Static | Anecdote	8.28	4.09	< 0.001*
Pre-intervention concern	0.87	36.54	< 0.001*

One-sample t tests—Change in concern versus 0				
Condition	*df*	*t*	*p*	95%CI
Static	130	− 3.16	0.002*	[− 7.74, -1.78]
Anecdote	134	2.46	0.02	[0.79, 7.21]
Static + Anecdote	149	0.52	0.60	[− 1.75, 2.99]
Narrative Visualization	115	0.73	0.47	[− 1.96, 4.27]

ANCOVA Results—Session 1 (Post-Risk ~ Condition + Pre-Risk)			
Coefficients	Estimate	*t* value	*p*
Intercept	25.90	14.18	< 0.001
Static | Static + Anecdote	− 2.80	− 1.29	0.20
Static | Narrative Visualization	3.01	1.30	0.19
Static | Anecdote	3.36	1.51	0.13
Pre-Intervention Risk	0.45	15.79	< 0.001*

One-sample t tests—Change in Risk versus 0				
Condition	*df*	*t*	*p*	95%CI
Static	130	3.30	0.001*	[3.23, 12.93]
Anecdote	134	8.57	< 0.001*	[10.25, 16.40]
Static + Anecdote	149	3.30	0.001*	[2.58, 10.27]
Narrative Visualization	115	6.19	< 0.001*	[9.53, 18.49]

*indicates significance of *p* < 0.003

Next, we ran a series of one-sample t-tests to determine if participants’ change in concern (posttest concern score—pretest concern score) was significantly different from 0 in each condition. All model output is available in Table 2. We found that participants in the Static Condition were *less* concerned after viewing the intervention materials. This decrease is reminiscent of a "backfire effect" (Lewandowsky et al., 2012). Past research has shown that attempts to debunk misinformation often lead to the denial of that information and further polarize opinion (Cook & Lewandowsky, 2016). When people do not have access to information about the data-generating process they may (understandably) find it difficult to understand the data, which may ultimately lead to *reduced* concern. However, it is worth noting that there is currently debate as to whether such backfire effects are robust (see Swire-Thompson et al., 2020 for a review).

Using persons as effect sizes to achieve converging evidence (Grice et al., 2020), we examined the proportion of participants per condition whose concern increased after viewing the intervention materials (Fig. 5b). The Static Condition was least effective at increasing people’s COVID-19 concern. The proportion of people who increased their concern was similar across the other conditions, with the Anecdote Condition having the largest proportion of participants whose concern increased.

*Change in Perceived Risk.* It is important to note that all participants were given explicit instructions on the risk of dining with 10 people, regardless of condition. Thus, the risk question asked post-intervention - “What is the risk that at least one person at a Thanksgiving table with 10 people has COVID-19?” - was a simple recall task. We compared post-intervention perceived risk between conditions using an ANCOVA, controlling for pre-intervention risk perception as a covariate. Pre-intervention perceived risk predicted post-intervention perceived risk (*p* < 0.001); however, there was no effect of condition on perceived risk (see Table 2).

One-sample t-tests with the test value as 0 (no change in perceived risk) indicated that perceived risk increased among all conditions after the intervention. Using persons as effect sizes to achieve converging evidence (Grice et al., 2020), we examined the proportion of participants per condition whose perceived risk increased after viewing the intervention materials (Fig. 5d). The Static Condition was the least effective at increasing perceived risk, although proportions were similar across conditions (see Table 2).

*Concern about Thanksgiving Plans*. After completing the intervention, participants were also asked to rate how concerned they were with their current Thanksgiving plans on a scale of 0–100 (*not concerned*—*extremely concerned*). A one-way ANOVA did not find a difference in concern between the four conditions (Mean Ratings: Static Condition = 40.09, Narrative Visualization Condition = 39.81, Static + Anecdote Condition = 42.66, Anecdote Condition = 43.91; see Table 3).

**Table 3:** Behavioral Intentions Reported in Sessions 1 and 2

Pre-Thanksgiving Intentions		
A. ANOVA—Comfort with Current Thanksgiving Plans		
	F (3,528)	*p*
Overall Model	0.7	0.55

Post-Thanksgiving Intentions		
	$\chi$ 2	*p*
Proportion of participants who changed Thanksgiving plans by condition	3.13	0.37
Proportion of participants who decreased Thanksgiving attendees by condition	1.37	0.71
Proportion of participants who planned to spend holidays with others by condition	3.28	0.35
Proportion of participants who planned to spend holidays with fewer people by condition	6.07	0.10

#### Post-thanksgiving follow-up survey

We next examined the data from the participants who returned for Session 2 and completed the Post-Thanksgiving survey. We examined data from participants who indicated that they planned to spend Thanksgiving with others during the Pre-Thanksgiving session (*n* = 407) as we considered those participants to be the least risk-averse.

*Holiday Plans*. Participants were asked to report information on how they spent their Thanksgiving holiday. We compared the proportion of participants who reported making last-minute changes to their Thanksgiving plans (*n* = 80) between conditions with a Chi-square test and found no differences between conditions (see Table 3). Participants who *did* change their Thanksgiving plans were asked whether the change increased, decreased, or did not impact the number of people at their gathering. The proportion of participants who  spent the holiday with fewer people was compared between conditions, and there was no significant difference between conditions (see Table 3).

Participants were also asked whether they planned to spend the December/January holidays alone or with others. We excluded  participants who responded “Unsure” (*n* = 22) and compared the proportion of participants who planned to spend the holidays with others between the four conditions using a Chi-square test. There was no significant difference between groups, with 54% of the Static + Anecdote Condition, 59% of the Narrative Visualization Condition, 62% of the Static Condition, and 51% of the Anecdote Condition, indicating that they planned to spend the holidays with others (see Table 3).

Participants were also asked whether the COVID pandemic had changed their usual holiday plans. We compared the proportion of participants from each condition who indicated that they planned to spend the holidays with fewer people than usual using a Chi-square test. There was no significant difference between conditions, with 69% of the Static + Anecdote Condition, 80% of the Narrative Visualization Condition, 69% of the Static Condition, and 80% of the Anecdote Condition indicating that they planned to spend the holidays with fewer people than usual (see Table 3).

*Concern Ratings*. We also asked participants how concerned they were about getting COVID or their family getting COVID at holiday gatherings. Their responses to these two items were averaged to create a composite concern score. We compared average concern between conditions with a one-way ANOVA and found no significant differences in concern between conditions (see Table 4). We did not use an ANCOVA as in Session 1 since we did not have participants rate their concern about the December holidays pre-intervention.

**Table 4:** Concern and Change in Perceived Risk in Session 2

	F (3403)	*p*
Overall Model	1.08	0.36

ANCOVA Results—Session 1 (Post-Risk ~ Condition + Pre-Risk)			
Coefficients	Estimate	*t* value	*p*
Intercept	28.76	10.74	< 0.001
Static | Static + Anecdote	1.43	0.44	0.66
Static | Narrative Visualization	− 0.31	− 0.09	0.93
Static | Anecdote	0.65	0.20	0.84
Pre-Intervention Risk	0.17	3.88	< 0.001*

One-sample t tests—Change in Risk versus 0				
Condition	df	*t*	*p*	95%CI
Static	103	1.29	0.20	[− 2.18, 10.30]
Anecdote	103	1.49	0.14	[− 1.61, 11.36]
Static + Anecdote	107	1.74	0.08	[− 0.77, 12.03]
Narrative Visualization	90	1.99	0.05	[0.02, 13.54]

*indicates significance of *p* < .003

*Change in Perceived Risk*. Participants asked to report the risk (0–100%) that at least one person at a table of 10 would have COVID at a Thanksgiving dinner. We investigated change in perceived risk using an ANCOVA model with condition as the predictor and pre-intervention perceived risk as the covariate. We found no significant differences between groups (see Table 4). One-sample t-tests compared the change in perceived risk from pre-intervention to Session 2 to 0 for each condition, and we did not find evidence for significant increases in perceived risk for any of the conditions (see Table 4).

#### Exploratory analyses

**Fig. 7:**
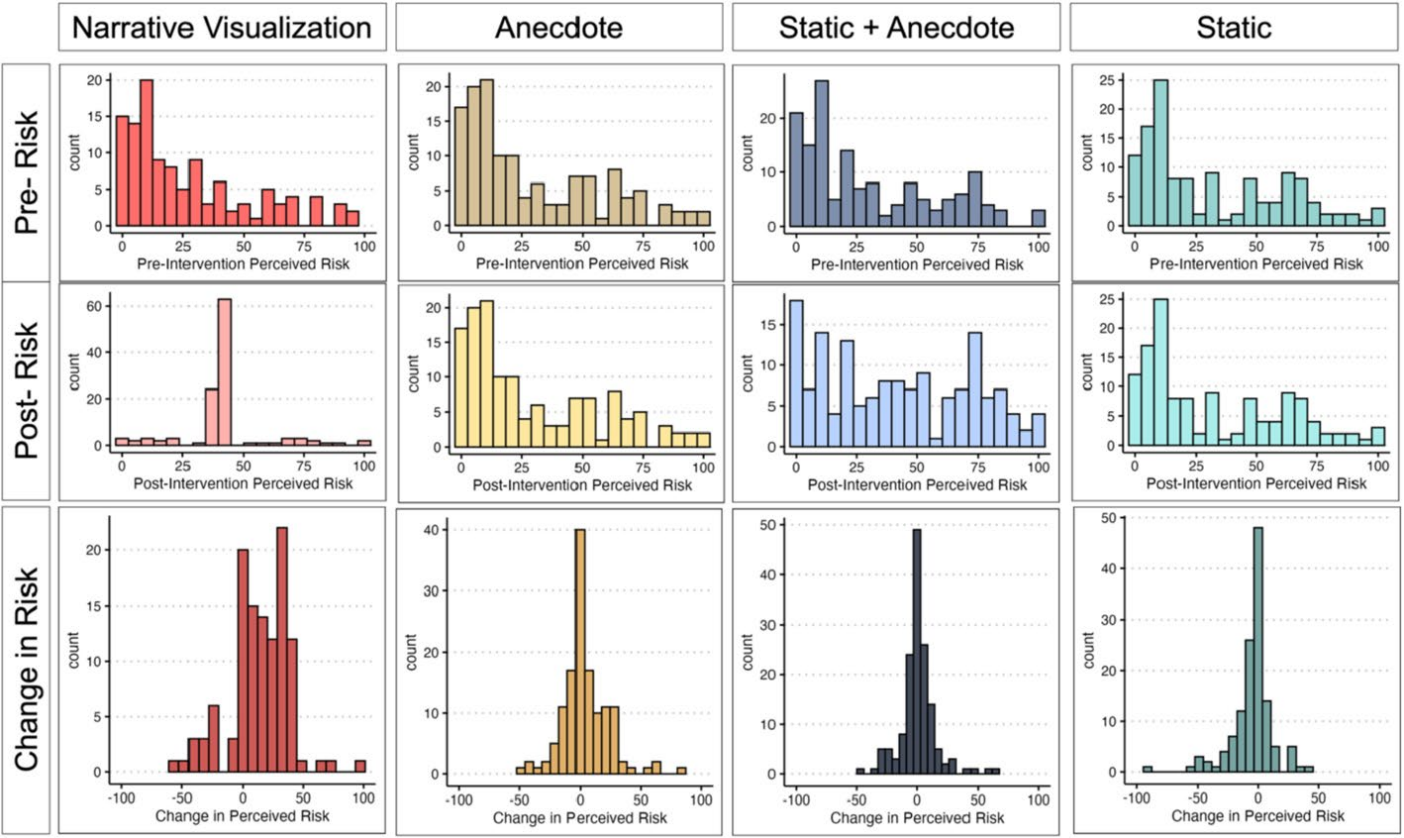
Distributions of Perceived Risk

We ran a series of exploratory analyses to investigate (1) the factors leading to participants’ decisions and concern relating to real-world plans, and (2) the factors leading participants to change their concern/perceived risk after viewing the intervention materials. Since these analyses are exploratory, we decided to be more lenient with our interpretation of significance, setting our alpha level to *p* < 0.05 (Fig. 7).

**Fig. 8:**
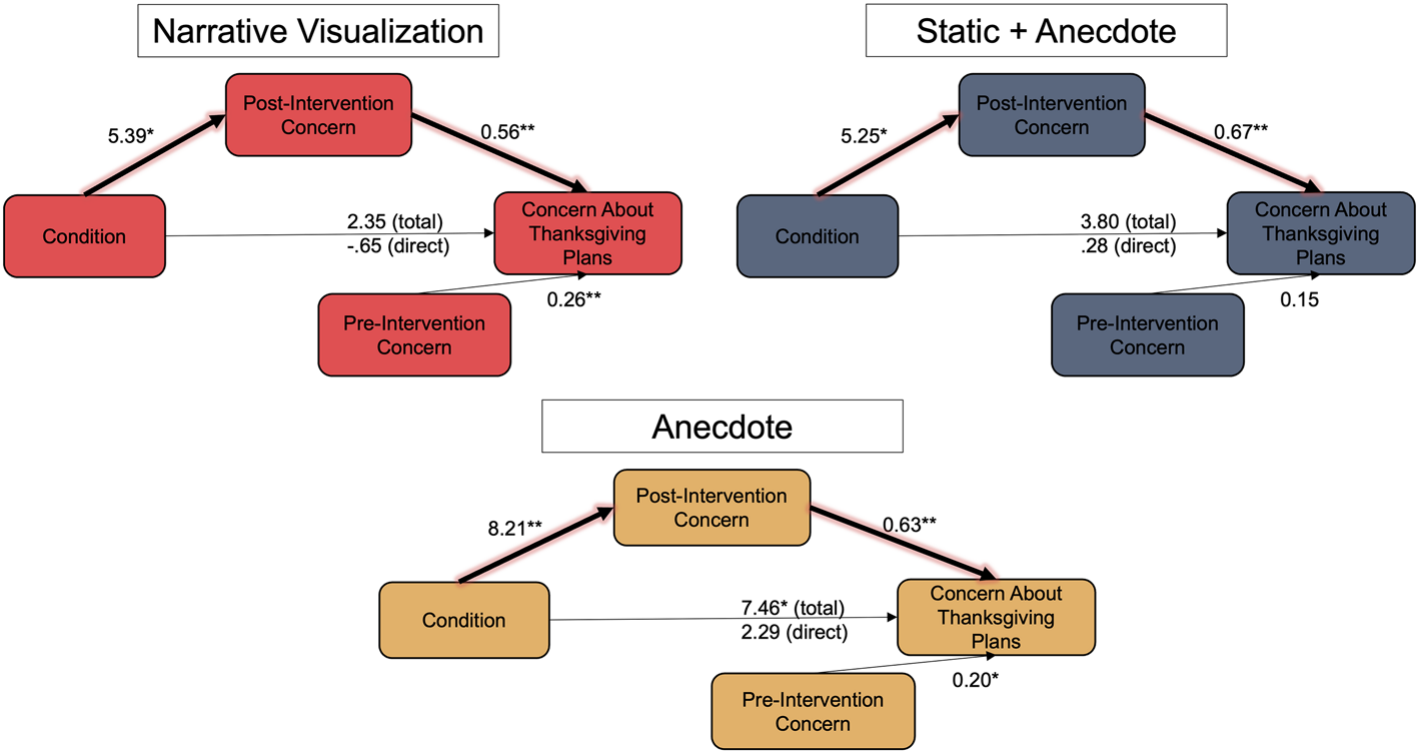
Exploratory Mediation Models. *Note*: For each model, the Static Condition is used as the reference group for each Condition comparison. *p < .05, *p < .001

*Exploratory Mediation Models*. As reported above, we did not find an effect of condition on participants’ real-world behavioral intentions. Thus, we explored the possibility that post-intervention concern mediated the relationship between condition and real-world behavioral intentions and decision-making. We ran a series of mediation analyses with condition as the predictor, pre-intervention concern as a covariate, post-intervention concern as the mediator, and real-world decisions/concern about plans as the outcome variables. For each model, only two conditions were included, with the Static Condition as the reference group, considering that the Static Condition showed a backfire effect in Session 1. Using the Static Condition as the reference condition also allowed us to investigate the impact of adding an anecdote (Static + Anecdote) or explanation of the data (Narrative Visualization) to data on concern about plans/behavioral intentions. The specific outcome variables used were: (1) concern about current Thanksgiving plans (collected in Session 1), (2) whether participants actually changed their Thanksgiving plans after the intervention (collected in Session 2), and (3) whether participants planned to spend the December holidays with others (collected in Session 2). All of the mediation models were run using the PROCESS macro in R (Hayes, 2017), with the Model 4 specification. We did not find any evidence that post-intervention concern mediated the relationship between condition and whether participants actually changed their Thanksgiving plans, nor whether participants planned to spend the December holidays with others. However, we found evidence for a full mediation of post-intervention concern on the relationship between condition and participants’ concern with their current Thanksgiving plans in Session 1. This evidence was present for all three models comparing the Static Condition to the rest of the intervention conditions (see Fig. 8). These findings suggest that relative to the Static Condition, the other interventions led to increased concern, which in turn increased participants’ concerns about their Thanksgiving plans. Given that all participants planned to celebrate Thanksgiving with others, this suggests that participating in the three interventions impacted their risk perception of real-world decisions in a positive way relative to the Static Condition.

*Predicting Increased Concern*. As illustrated by Fig. 6, it was surprising to us that many participants’ concerns did not increase after viewing their respective intervention. Thus, we ran an exploratory analysis examining the factors predicting whether one’s concern increased after viewing an intervention. We ran a logistic regression with political partisanship (coded as *right*, *left*, or *other,* with *right* as the reference group), numeracy based on the subjective numeracy scale, condition (with the Static Condition as the reference group), and pre-intervention concern as predictors, and whether participants’ concern increased (1) or stayed the same/decreased (0) as the outcome variable:

$$Increased \,Concern \left(0 \,or \,1\right) \sim Condition+Partisanship+Numeracy+Pre-Concern$$

We found evidence that participants in all three conditions were more likely to increase their level of concern than the Static Condition, and that participants who identified with the political left (i.e., Democrat or Lean Democrat) were more likely to increase their level of concern than participants who identified with the political right (i.e., Republican or Lean Republican) (see Table 5). There was no effect of numeracy nor pre-intervention concern on whether participants’ concern increased.

**Table 5:** Factors associated with concern increase—logistic regression output

	Estimate	Std. Error	*z* value	*p*
Intercept	− 1.10	0.49	− 2.24	0.03
Static + Anecdote versus Static	0.60	0.25	2.40	0.02*
Narrative Visualization versus Static	0.64	0.27	2.38	0.02*
Anecdote versus Static	0.80	0.26	3.12	0.002*
Partisanship: Left versus Right	0.53	0.23	2.30	0.02*
Partisanship: Other versus Right	0.07	0.26	0.24	0.80
Numeracy	0.03	0.08	0.39	0.70
Pre-intervention concern	− 0.003	0.003	− 0.99	0.32

### Discussion

The goal of Study 1 was to examine whether narrative visualizations are an effective tool for communicating cumulative risk, as well as how other interventions such as the use of anecdotes and static data visualizations influenced perceptions of cumulative risk. We collected these data in a unique scenario where participants reasoned about the real-world risks associated with dining with others during the COVID pandemic in 2020. Our most notable finding was a data backfire effect in the Static Condition in Session 1, suggesting that viewing the cumulative risk data led to *decreased* concern about the risk of contracting COVID at Thanksgiving dinner. It is important to note that this lack of concern was not due to a misunderstanding of the risk information presented to participants. Indeed, participants across all conditions were presented with the same cumulative risk of contracting COVID from a 10-person dinner, and there were no significant differences in perceived risk post-intervention between conditions. This suggests that although participants in the Static Condition perceived risk similarly to the other conditions, they were simply less concerned about the risk.

Four weeks after the intervention, we did not find statistically significant differences between conditions in terms of their behavioral intentions. A similar proportion of participants reported changing their Thanksgiving plans after the intervention among conditions, and the proportion of participants who planned to spend the holidays alone or with fewer people was also similar across conditions. We found little evidence that the effects of the intervention were long lasting, as concern ratings and change in perceived risk were similar across conditions at the Post-Thanksgiving survey stage. However, in an exploratory mediation analysis we found evidence that relative to the Static Condition, participating in the Static + Anecdote, Narrative Visualization, and Anecdote Condition was associated with greater general concern which was associated with increasedconcern about one’s Thanksgiving plans to dine with others. This provides some evidence that participants connected the data presented to them to their real-world decisions and intentions.

Lastly, we found that a large number of participants did not report any increase in concern from pre- to post-intervention. In an exploratory analysis, we found evidence that participants who politically leaned to the left were more likely to increase their concern from pre- to post-intervention compared to those who leaned right. This suggests that left-leaning participants may have been more inclined to update their beliefs in the presence of new information.

The most notable finding from Study 1 was that viewing data by itself led to a backfire effect—where participants were less concerned about a risk after viewing a bar graph illustrating cumulative risk. However, viewing static data in conjunction with an anecdote prevented this data backfire effect, as did viewing a narrative visualization. In Study 2, we further explore why these interventions did not lead to a backfire effect by examining how the various interventions influenced trust and understanding of the data.

## Study 2

Study 1 showed that viewing a static visualization of cumulative risk was associated with increased risk perception but *decreased* concern from pre- to post-intervention. This backfire effect was not present inthe other conditions where participantsviewed either an anecdote by itself, a static visualization with an anecdote, or a narrative visualization. This provides promising evidence that interventions can include data without leading to backfire effects on concern. It is also encouraging that the Narrative Visualization Condition did not lead to a data backfire effect even though this intervention did not rely on emotional appeal like the other conditions (Anecdote/Static + Anecdote Conditions), suggesting that narrative visualizations may be an effective tool for communicating risks. Why didn’t the Narrative Visualization Condition lead to the data backfire effect that occurred in the Static Condition? It is possible that people who viewed the narrative visualization were able to obtain a better understanding and/or had more trust in the data because of the additional information provided by the walkthrough, compared to the static visualization. The aim of Study 2 was to replicate our findings from Study 1 while examining whether the additional information provided by the narrative visualization affected trust, subjective understanding, or both. We also examined how these variables were influenced by the presence of anecdotes, which do not include information about how the data were generated, but also mitigated the backfire effect in Study 1. All data, materials, and code are available at https://osf.io/k43ev/?view_only=67cc6401b06946889ac499fc19afec2f.

### Methods

#### Participants

We recruited 1592 US participants (806 female; age M(SD) = 44.67(16.04)) from Prolific between January 6, 2022 and February 5, 2022. Participants received an average pay of $17.04/hr (USD), and the median completion time for the study was 5 min and 36 s. All procedures were determined to be exempt by the University of Michigan IRB.

#### Design

As in Study 1, participants were randomly assigned to one of four conditions in which they viewed either a static visualization (Static Condition, *n* = 399), the same static visualization combined with an anecdote (Static + Anecdote Condition, *n* = 388), an anecdote presented without a visualization (Anecdote Condition, *n* = 404), or a narrative visualization (Narrative Visualization Condition, *n* = 401).

#### Materials

*Pre-Intervention Materials*. Data for Study 2 were collected in January and February of 2022, so we made several changes to the Study 1 materials to reflect developments  that had occurred in the world (e.g., the development of COVID-19 vaccines) during the time between the two studies. To account for changes in the COVID-19 pandemic and access to vaccines, participants were asked to make judgments about their hypothetical Thanksgiving plans during a pandemic with a fictional disease for which there was not yet a vaccine (as there were no vaccines available for COVID-19 when the Study 1 data were collected). All items with exact wording are included in the Supplementary Materials. All participants were presented with this scenario:

Imagine the following scenario: a **new respiratory disease** emerged in your community. This new disease is **highly contagious**, and there is **no vaccine available** yet. The Fall & Winter holiday season is approaching, and public health officials recommend that people do not gather during the holidays, but your local government has not placed any restrictions on gathering.

Please answer some questions about what your Thanksgiving plans would have been this year **if you were living through the above hypothetical scenario.**

Next, participants were asked who they would have Thanksgiving dinner with in this hypothetical scenario (i.e., alone/with people who live with you, extended family, friends/neighbors, strangers). They were also asked to indicate how concerned they would be about contracting the disease at Thanksgiving dinner and how concerned they would be that someone else at their Thanksgiving dinner would contract the disease (slider scales from 0 (*not concerned at all*)—100 (*extremely concerned*) for each item). To assess understanding of risk, participants were asked “If 4% of the population has the disease, what is the risk that at least one person at a Thanksgiving table with 10 people has the disease?” which they answered with a slider scale from 0 to 100%.

**Fig. 9:**
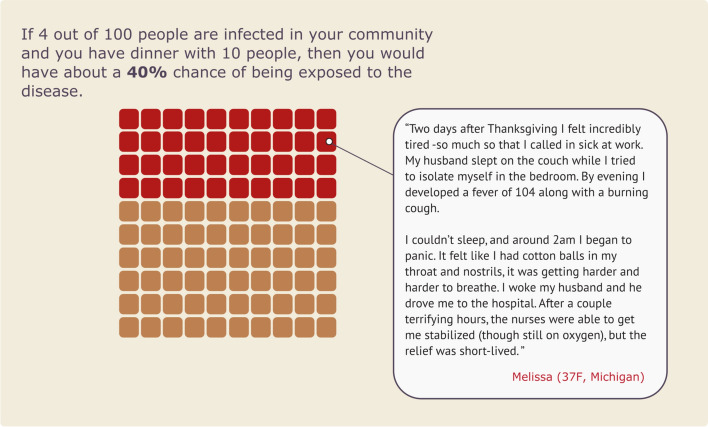
Static + Anecdote Condition materials for Study 2. *Note.* The Static Condition materials contained the same icon array without the anecdote. The Anecdote Condition materials contained an expanded version of the anecdote with no visualized data (see Supplementary Materials)

**Fig. 10:**
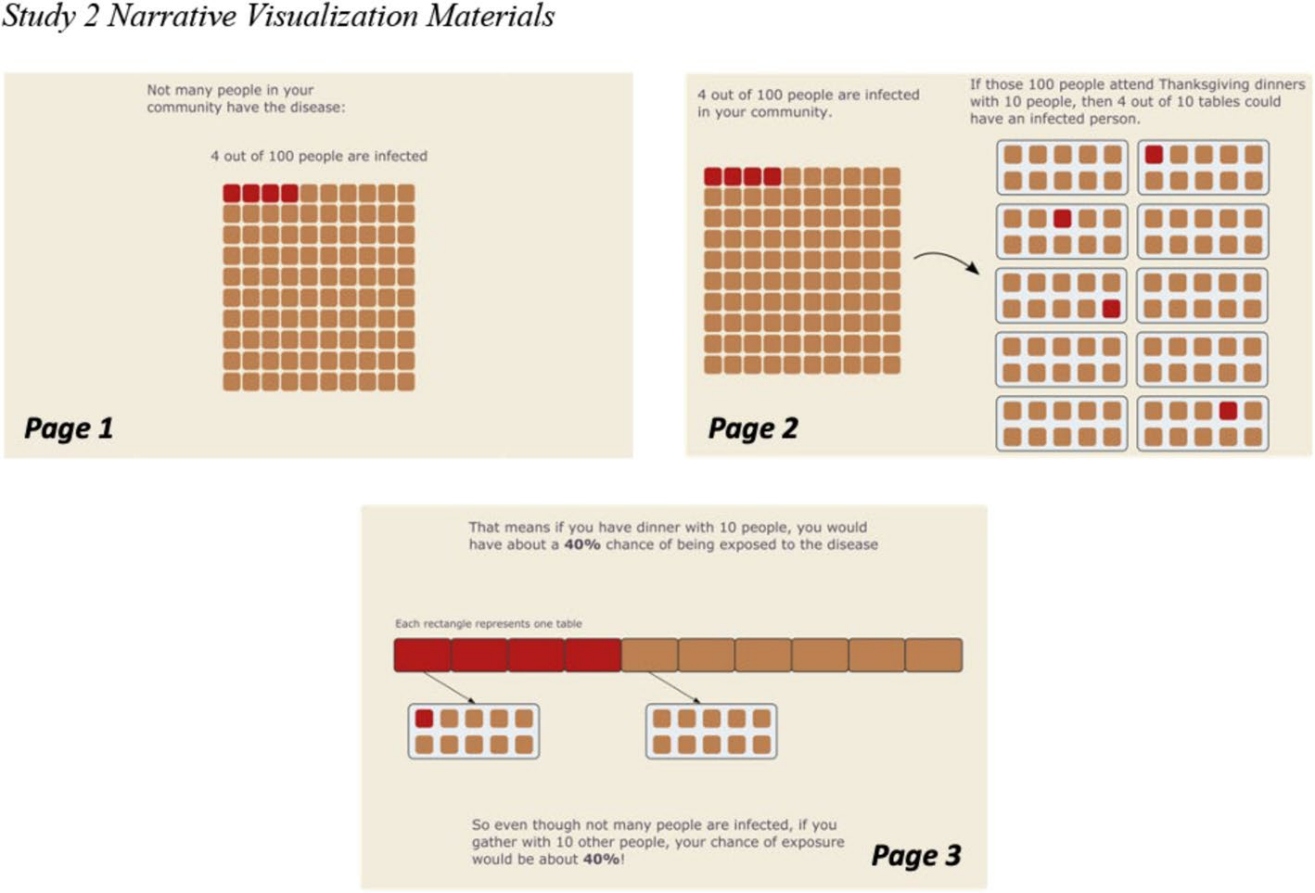
Study 2 Narrative Visualization Materials. *Note.* Participants viewed three consecutive pages which explain step-by-step how the risk of exposure to the disease is about 40% in the study scenario (i.e., a Thanksgiving dinner with 10 people taking place in a community where 4 out of 100 people are infected)

*Intervention Materials*. We made several refinements to the Study 1 intervention materials for Study 2. In the Static and Static + Anecdote Conditions of Study 2, we presented the risk information as an icon array (Fig. 9), rather than the bar graph from Study 1 (Fig. 3). Icon arrays are thought to increase understanding of risk, particularly for individuals with low numeracy (Okan et al., 2015). We made this change to improve the robustness of the Static Condition as a baseline. In addition, the narrative visualization in Study 1 used seven consecutive displays (Fig. 1), while the static intervention used a single static visualization (Fig. 3). To reduce the difference in time on task between these two interventions, we shortened the narrative visualization to just three displays (Fig. 10). Finally, the anecdote intervention in Study 1 did not include the quantitative risk data presented in the other interventions. To reduce differences between interventions, we modified the final paragraph of the anecdote to include the critical quantitative conclusion below (bold text was added for Study 2):

I’m so scared, and words can’t describe the guilt I feel. I know I’m just one person, but if you take anything away from this story please STAY HOME. **The pandemic has been hard for everyone, and we all miss our families, but the risks can be much higher than we realize. I found out afterward that (according to the CDC) even though only about 4 out of 100 people are infected, there was about a 40% chance that someone at our dinner of 10 was infected.** So please don’t risk something like this happening to you or your family. Stay home not just to protect yourself, but to protect your loved ones too.

We also modified the anecdote to remove any language that referred to COVID specifically. The full anecdote used in Study 2 is available in the Supplementary Materials.

*Post-Intervention Survey Materials*. After viewing the intervention materials, we asked participants to rate their concern about contracting the disease at Thanksgiving dinner for themselves and for others on slider scales of 0 (*not concerned at all*) to 100 (*extremely concerned*). We also asked them how they felt about the hypothetical Thanksgiving plans they reported pre-intervention with a slider scale from 0 (*not concerned at all*) to 100 (*extremely concerned*).

To measure self-rated understanding, we asked participants to rate their level of agreement with the following statement from 0 to 100: “After viewing the data, I understand why a Thanksgiving dinner with 10 people has about a 40% chance of exposure to the disease.” To measure trust, we asked them to rate their level of agreement on scales of 0–100 with the following items:

1. I feel like the data are intended to accurately portray the risks of the new disease.
2. I feel like the data do accurately portray the risks of the new disease.

Lastly, participants provided demographic information, including numeracy and political partisanship data as in Study 1.

### Procedure

The procedure for Study 2 was the same as for Study 1 except that participants were not invited for a follow-up survey four weeks later.

### Results

As in Study 1, given that we present the results of multiple tests, we used a Bonferroni correction to reduce the likelihood of Type I error. Therefore, in the following analyses comparing concern, trust, and understanding between conditions, we lowered our significance level to *p* < 0.007, as  7 of our tests did not correct for multiple comparisons ($\alpha =\frac{.05}{7}= .007$).

#### Exclusion criteria

The Post-Intervention Survey contained two attention check items:

1. According to the data at the end of the story, what is the approximate risk that you will be exposed to the disease if 4 out of 100 people in your community are infected and you have dinner with 10 people? (40%, 4%, 10%, or 14%?)
2. In the hypothetical scenario you read at the beginning of the study, was a vaccine available for the disease? (Yes, one vaccine, Yes, multiple vaccines, or No).

Participants were excluded from the analysis if they gave an incorrect response on one or both attention check questions (*n* = 156). Of participants who passed both attention check items, only 12% of participants (*n* = 173) stated that they would eat dinner with people outside of their home, so we were unable to divide the data based on dinner intentions as we did for Study 1.

#### Post-intervention concern

**Fig. 11:**
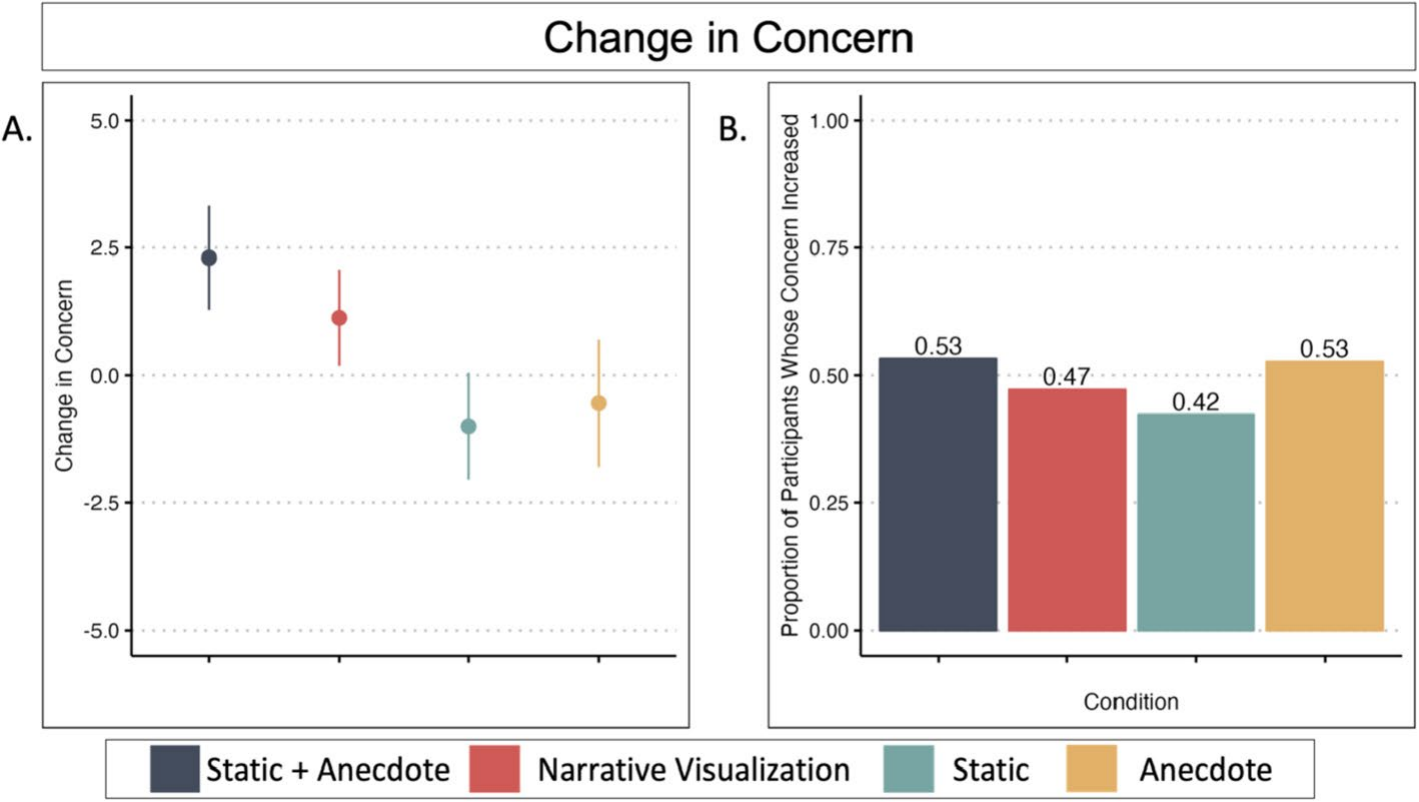
Study 2 Changes in Concern and Perceived Risk by Condition. *Note.* Figure 11a illustrates condition means and standard error. A change in concern of zero indicates no change; positive scores indicate increased concern, and negative scores indicate decreased concern. Figure 11b shows the proportion of participants whose concern increased in each condition

All model output is available in Table 6. To compare post-intervention concern across intervention conditions, we ran an ANCOVA with condition as the predictor, pre-intervention concern as the covariate, and post-intervention concern as the outcome variable of interest (see Fig. 11a). Unlike Study 1, there were no significant differences between conditions.

**Table 6:** Change in Concern from Pre- to Post-Intervention in Study 2

ANCOVA Results—Study 2 (Post-Concern ~ Condition + Pre-Concern)				
Coefficients	Estimate	*t* value	*p*	
Intercept	11.99	7.68	< 0.001	
Static | Static + Anecdote	3.34	2.29	0.02	
Static | Narrative Visualization	2.35	1.63	0.10	
Static | Anecdote	0.92	0.63	0.53	
Pre-intervention concern	0.80	45.53	< 0.001*	

One-sample t tests—Change in concern versus 0				
Condition	df	*t*	*p*	95%CI
Static	356	− 0.95	0.34	[− 3.06, 1.06]
Anecdote	354	− 0.44	0.66	[− 3.01, 1.91]
Static + Anecdote	350	2.26	0.02	[0.30, 4.31]
Narrative Visualization	372	1.20	0.23	[− 0.73, 2.99]

*Indicates significance of *p* < .007

**Fig. 12:**
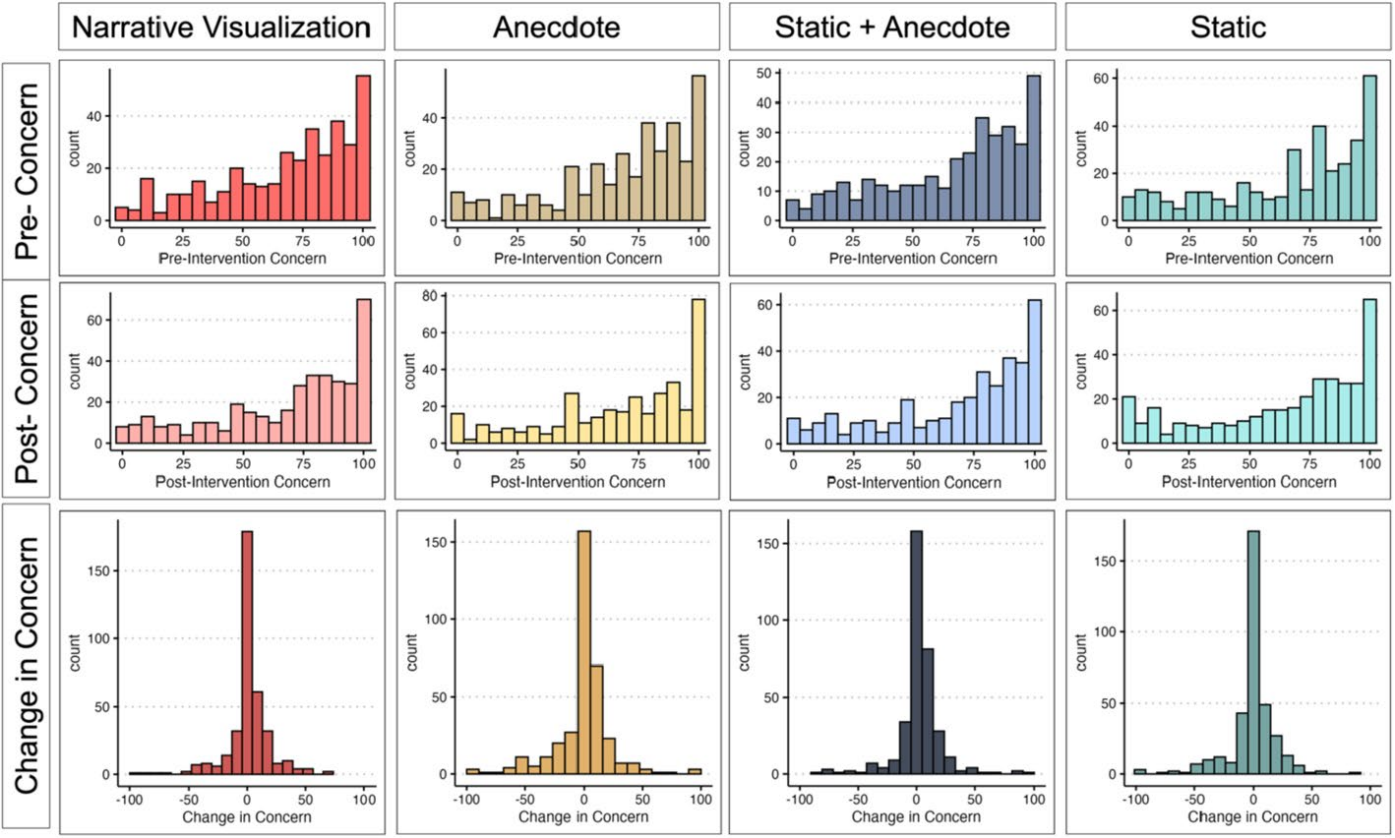
Distributions of concern scores

Next, we ran a series of one-sample t-tests with the comparison value as 0 to determine if change in concern significantly increased or decreased in each condition. After correcting for multiple tests, we did not find evidence for a significant increase or decrease in participants’ concern in any of the conditions. Using persons as effect sizes to achieve converging evidence (Grice et al., 2020), we examined the proportion of participants per condition whose concern increased after viewing the intervention materials (Fig. 11b). Similar to Study 1, the Static Condition was least effective at increasing people’s COVID-19 concern. Distributions of pre-intervention, post-intervention, and change in concern scores are shown in Fig. 12.

#### The effect of condition on trust and understanding

Our primary question in Study 2 was whether the narrative visualization intervention and Static + Anecdote Conditions prevented a data backfire effect because participants felt that they understood and trusted the data more than in the Static Condition. We first tested our hypothesis that viewing the narrative visualization would improve understanding and trust compared to the Static Condition.

**Fig. 13:**
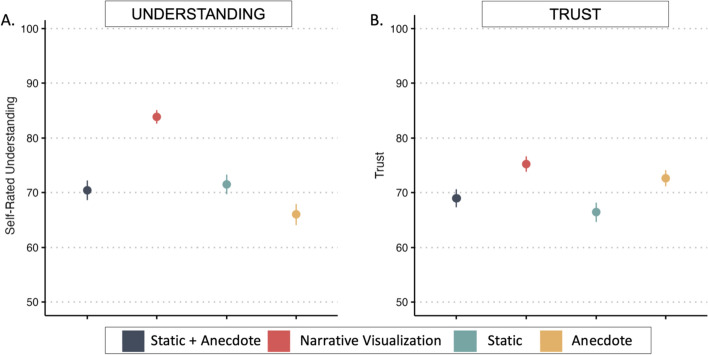
Study 2 self-rated understanding and trust by condition. *Note.* Figures 13a–b illustrate mean and standard error for self-rated understanding and trust by condition

Self-rated understanding was measured through response to the item “After reading the story, I understand why a Thanksgiving dinner with 10 people has about a 40% chance of exposure to the disease.” To examine the role of condition on self-rated understanding, we ran a one-way ANOVA with Condition as the predictor and understanding as the outcome variable. The model was significant overall (see Fig. 13a; Table 7) and post hoc tests with Tukey HSD indicated that participants in the Narrative Visualization Condition reported greater understanding than those in the Static + Anecdote Condition, the Anecdote Condition, and the Static Condition.

**Table 7:** Trust and understanding by condition in Study 2

Study 2—Understanding		
ANOVA—Understanding by Condition		
	F (3,1432)	*p*
Overall Model	21.16	< 0.001*

Tukey HSD Significant Pairwise Comparisons			
	*M*_diff_	*p*_adj_	95%CI
Narrative Visualization | Static	13.48	< 0.001*	[7.37, 19.59]
Anecdote | Static + Anecdote	− 4.41	0.26	[− 10.59, 1.77]
Anecdote | Narrative Visualization	− 17.89	< 0.001*	[− 23.98, − 11.80]
Static | Static + Anecdote	1.08	0.97	[− 5.09, 7.26]
Static | Narrative Visualization	− 12.39	< 0.001*	[− 18.48, − 6.31]
Anecdote | Static	− 5.49	0.10	[− 11.65, 0.67]

Study 2—Trust		
ANOVA—Trust by Condition		
	F (3,1432)	*p*
Overall Model	6.23	< 0.001*

Tukey HSD Significant Pairwise Comparisons			
	*M*_diff_	*p*_adj_	95%CI
Narrative Visualization | Static	6.28	0.02	[0.59, 11.97]
Anecdote | Static + Anecdote	3.68	0.36	[− 2.08, 9.43]
Anecdote | Narrative Visualization	− 2.60	0.64	[− 8.28, 3.07]
Static | Static + Anecdote	− 2.53	0.67	[− 8.28, 3.22]
Static | Narrative Visualization	− 8.81	< 0.001*	[− 14.47, − 3.14]
Anecdote | Static	6.20	0.03	[0.47, 11.94]

*Indicates significance of *p* < .007

Trust was calculated by averaging participants’ responses to the two items: (1) "I feel like the story is intended to accurately portray the risks of the new disease" and (2) "I feel like the story does accurately portray the risks of the new disease". We again ran a one-way ANOVA with condition as the predictor and trust as the outcome variable. The model was significant overall (see Fig. 13b; Table 7) and post hoc tests with Tukey HSD indicated that participants in the Narrative Visualization Condition reported greater trust than those in the Static Condition.

#### Mediation analysis

The primary goal of Study 2 was to investigate the possible mechanisms that allowed participants in the Narrative Visualization Condition to view the data without experiencing a data backfire effect (as seen in  the Static Condition). As such, we investigated the impact of intervention condition on participants’ self-reported understanding of the data and trust in the data.

#### Mediation analysis—comparing narrative visualization and static conditions

**Fig. 14:**
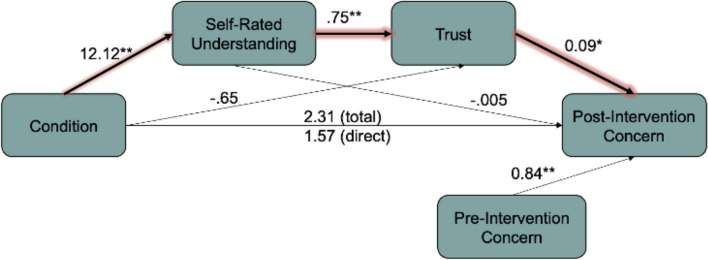
Study 2: Mediation model comparing the Narrative Visualization Condition to the Static Condition. *Notes:* *p < 0.05; **p < 0.001. Thick lines show the significant indirect pathway from condition to post-intervention concern via understanding and trust

We conducted a mediation analysis using the PROCESS macro in R (Hayes, 2017) to determine whether the impact of the narrative visualization on post-concern ratings was serially mediated by understanding and trust in the data (Model 6 in the PROCESS macro). We ran a mediation analysis with understanding and trust as mediators on the effect of the narrative visualization (IV) on post-intervention concern (DV) (Fig. 14). To isolate the specific effect of the narrative visualization, we compared the narrative visualization intervention to the static intervention. Pre-intervention concern was included as a covariate.

*The Effect of Condition on Understanding and Trust*. We ran a serial model with the idea that understanding affects trust. The outcome is shown in Fig. 14. We found evidence for an independent effect of condition on understanding: participants in the Narrative Visualization Condition reported greater understanding compared to participants in the Static Condition (*b* = 12.12, *p* < 0.001, 95% CI = [8.00, 16.24]). There was also an independent effect of understanding on trust, such that greater understanding was associated with higher trust (*b* = 0.75, *p* < 0.001, 95% CI = [0.70, 0.79]). However, there was no significant independent effect of condition on trust itself (*b* = − 0.65, *p* = 0.64, 95% CI = [− 3.41, 2.10]).

*Predicting Post-Intervention Concern*. There was an independent effect of trust in the data on concern about disease transmission post-intervention (*b* = 0.09, *p* = 0.01, *95%*CI = [0.02, 0.17]). However, reported understanding did not independently predict post-intervention concern (*b* = − 0.005, *p* = 0.90, 95% CI = [− 0.08, 0.07]). There was also no evidence of a direct effect of condition on post-intervention concern (*b* = 1.57 *p* = 0.25, 95% CI = [− 1.15, 4.30]).

*Mediating the Relationship Between Condition and Concern*. We did not find evidence for an indirect effect of condition on concern through understanding (indirect effect = − 0.05, SE = 0.44, 95% CI = [− 0.94, 0.82]) or trust (indirect effect = − 0.06, SE = 0.15, 95% CI = [− 0.38, 0.21]). However, the data suggested that condition had an indirect effect on concern through both self-rated understanding *and* trust (indirect effect = 0.85, SE = 0.37, 95% CI = [0.20, 1.64]). These findings suggest a full serial mediation of understanding and trust on the relationship between condition and post-intervention concern.

#### Mediation analysis—comparing static + anecdote and static conditions

In Study 1, we found evidence of a data backfire effect, in which participants in the Static Condition reported feeling *less* concerned after seeing the data. Even though participants in the Static + Anecdote and Narrative Visualization Conditions also viewed data, they did not experience the same effect. Therefore, we thought it would be helpful to examine the mechanisms that prevented the data backfire effect in the Static + Anecdote Condition. We again conducted a mediation analysis to examine the effect of condition, understanding, and trust on post-intervention concern.

*The Effect of Condition on Understanding and Trust*. We did not find an independent effect of condition on reported understanding (*b* = −1.14, *p* = 0.65, 95% CI = [− 5.95, 3.67]); however, participants in the Static + Anecdote Condition reported feeling higher levels of trust than participants in the Static Condition (*b* = 3.28, *p* = 0.02, 95% CI = [0.58, 5.97]). As in the previous analysis, there was an independent effect of understanding on trust; participants who reported greater understanding also reported greater trust in the data (*b* = 0.72, *p* < 0.001, 95% CI = [0.12, 0.22]).

*Predicting Post-Intervention Concern*. We found evidence for a direct effect of condition on post-intervention concern, such that participants in the Static + Anecdote Condition reported greater concern than the Static Condition (*b* = 3.15, *p* = 0.03, 95% CI = [0.40, 5.91]). There was also an independent effect of trust on concern, with participants reporting greater trust also reporting greater concern post-intervention (*b* = 0.08 *p* = 0.03, 95% CI = [0.007, 0.16]). There was no independent effect of reported understanding on post-intervention concern (*b* = 0.02, *p* = 0.63, 95% CI = [− 0.05, 0.08]).

**Fig. 15:**
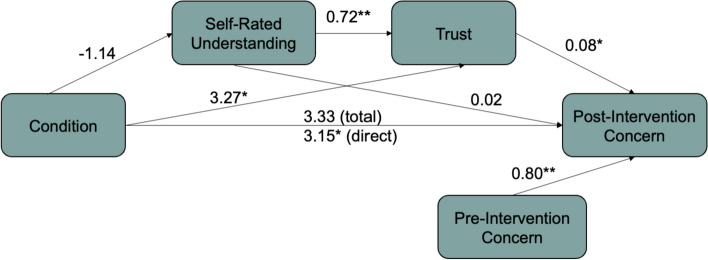
Study 2: Mediation model comparing the Static + Anecdote Condition to the Static Condition. Notes: *p < 0.05; **p < 0.001

*Mediating the Relationship Between Condition and Concern*. We did not find evidence for an indirect effect of condition on concern through understanding (indirect effect = − 0.02, SE = 0.11, 95% CI = [− 0.27, 0.21]) or trust (indirect effect = 0.27, SE = 0.20, 95% CI = [− 0.005, 0.74]). Unlike the previous analysis, we found no evidence of an indirect effect of condition on post-intervention concern through both understanding and trust (indirect effect = − 0.07, SE = 0.17, 95% CI = [− 0.45, 0.25] (see Fig. 15).

### Discussion

Unlike in Study 1, we did not observe statistically significant differences between intervention conditions in changes in concern. Study 2 results likely differed from Study 1 because of the differences in context between the two studies: Study 1 assessed concern about real-life plans during a time where COVID-19 was much less understood, while Study 2 asked about concern for hypothetical plans and was administered after the virus was better understood and a vaccine was widely available. Additionally, Study 2  included data from all the participants, whereas Study 1 exclusively used data from participants who were the least risk averse (indicated they planned to spend Thanksgiving with people outside of their household).

The findings from Study 2 provide a mechanistic explanation for why participants in the Narrative Visualization Condition did not experience a backfire effect observed in  the Static Condition. Our data suggest that viewing a narrative visualization led to increased subjective understanding of data and greater trust. Most importantly, a mediation analysis indicated that viewing the narrative visualization increased concern relative to viewing a static visualization, and that this effect was produced through increasing understanding and trust in the data. Thus, the model shows that the narrative visualization helped people feel more confident in their understanding of the risk, which, in turn, increased their trust in the information, ultimately resulting in an increase in overall concern.

## General discussion

Helping the general public accurately reason about data, especially in contexts related to real-world decision-making, is an important goal of science communication. The current investigation builds on previous work examining how viewing quantitative data and reading affect-laden stories shape risk perception across various domains (Cutello et al., 2020; Fagerlin et al., 2005; Rakow et al., 2015; Tiede et al., 2022). In Study 1, we compared different methods for presenting cumulative risk data regarding the likelihood of contracting COVID during the 2020 holidays. We tested the effectiveness of narrative visualizations, a method of data communication that walks participants through how data are generated, as a scientific communication tool. We also examined the impact of static data visualizations and anecdotal evidence on risk perception and concern about the COVID pandemic. The context of study allowed us to investigate how these interventions affected individuals with suboptimal risk perception or concern, in that participants included in our data analysis planned to dine with people outside of their household during the 2020 American Thanksgiving holiday. We were also able to examine the effects of viewing the intervention materials on perceptions of real-world decisions given the longitudinal design of in Study 1. A key finding from Study 1 was that participants who viewed a static visualization of cumulative risk experienced a backfire effect—even though their *perceived risk *of dining with individuals outside of their home *increased *from pre- to post-intervention, their *concern* about contracting COVID from pre- to post-intervention actually *decreased*. This backfire effect was not present observed in  the Static + Anecdote, or the Narrative Visualization Conditions.

It is perhaps unsurprising that the presence of an anecdote in the Static + Anecdote Condition helped mitigate this backfire effect, given prior work demonstrating the potential for emotion-laden stories to increase perceived risk (Freling et al., 2020). Frequently, risk avoidance strategies rely on providing people with alarming stories. For instance, to avoid driving distracted or driving under the influence of drugs or alcohol, risk messaging relies on impressionable and emotional testimonials rather than quantitative data (Betsch et al., 2011; Janssen et al., 2013; Kim et al., 2017). Although anecdotes are often effective, they can be problematic. Typically, anecdotes rely on affect-based mechanisms activating heuristic thinking, rather than analytic mechanisms (Rodriguez et al., 2016). Consequently, people tend to *ignore* quantitative data and instead make decisions based on testimonials or anecdotes even when data provided are more representative.

Narrative visualizations, in contrast, do not rely on affective responses and directly address the data at hand. In Study 1, we found that viewing a narrative visualization explaining how the accumulated risk data were generated prevented a backfire effect. This suggests that in real-world contexts where perceived risk is suboptimal, narrative visualizations can influence people’s attitudes by communicating quantitative data, rather than relying on stories or testimonials. Why was the narrative visualization effective? Our mediation analysis in Study 2 indicated that the mechanisms driving this effect were understanding and trust. A serial mediation analysis revealed a full serial mediation of understanding and trust on the relationship between condition and post-intervention concern. It is perhaps unsurprising that the narrative visualization increased perceived understanding and trust; the narrative visualization explains the data-generating process in detail, providing a causal explanation for the data. Research suggests that providing coherent explanatory frameworks is associated with higher judgements of information credibility (Sloman, 1994; Thagard, 2007). Causal explanations are also more effective in correcting misinformation compared to providing people with corrective information (Lammers et al., 2020; Lewandowsky et al., 2012; Nyhan & Reifler, 2015; Seifert, 2002). Understanding and trust did not mediate the relationship between condition and concern when comparingthe Static + Anecdote and Static Conditions, suggesting that the Static + Anecdote Condition mitigated backfire through a mechanism other than increased trust and understanding.

### Implications for scientific communication

Our findings provide evidence that risks can be communicated as quantitative data in a manner that still increases people’s understanding and trust in data. These findings suggest that narrative visualizations may be an effective tool for combating misinformation about data. Over- and underestimation of risks are common types of misinformation. Underestimation of risk, as observed in the present studies, is particularly common, and it is difficult to convince individuals to follow public health guidelines when risks are underestimated. Overestimation of risks is also problematic. For example, vaccine-hesitant individuals overestimate the risk of vaccines and believe these risks are more common and serious than they are in reality. Most prior work on correcting misinformation, however, focuses on correcting inaccurate facts (e.g., the belief that vaccines cause autism) rather than correcting data. For contexts such as vaccine hesitancy, there is some evidence that corrections can backfire (Nyhan & Reifler, 2015). In Study 1, we demonstrate that correcting quantitative - and not just factual - misinformation can also lead to backfire when data are surprising. Furthermore, we show that causal explanations are effective not only for correcting misinformation about facts, but also about data.

Our results demonstrate that narrative visualizations can be more effective than static visualizations for communicating risk, particularly in situations like the COVID-19 pandemic. Beyond this scenario, we believe narrative visualizations have the potential to communicate about complex, unintuitive, or surprising data in various other contexts. In particular, narrative visualizations may be useful in contexts involving accumulation(e.g., exposure to environmental toxins, or repeated engagement in risky behaviors) or exponential growth (compound interest in the context of financial literacy, or the spread of wildfires in the context of public safety), and in explaining outcomes related to algorithmic decision-making (e.g., recidivism prediction; O’Neil, 2017). By interleaving visualizations with explanations of the underlying data-generating processes, narrative visualizations can help readers build connections between the story and rationale,consequently increasing their trust in the data.

However, trust is not always beneficial. For example, Padilla et al. (2022a, 2022b) found that viewing lower-complexity visualizations was associated with increased trust in the data; however, this led to poor decision quality. Similarly, McGinnies and Ward (1980) found that people were more persuaded by a source’s trustworthiness than by their expertise, and O’Brien et al. (2021) found that greater trust in science can make people vulnerable to believing pseudoscience. It is important to note that data visualization should not lead to blind trust. Instead, visualizations should optimize the presentation of data so that users can critically evaluate information (i.e., users should “calibrate” their trust: Elhamdadi et al., 2022a, 2022b; Han & Schulz, 2020). This goal assumes that the designer wants the user to accurately understand the data and not misinform (e.g., see *Ethical Interaction Theory*; Feltz & Cokely, 2024 for further discussion).

### Open questions

Our findings raise several questions for further exploration. First, we did not find an effect of condition as it pertained to real-world decision-making and perceptions of decisions (i.e., whether one changed their Thanksgiving or December holiday plans in Study 1). In Study 1, we ran an exploratory analysis to investigate whether post-intervention concern mediated the relationship between condition and real-world outcomes. We found evidence that,compared to the Static Condition, participants in the three other intervention conditions (Static + Anecdote, Narrative Visualization, and Anecdote) reported increased concern, which was associated with increased concern about their  own Thanksgiving plans. These findings suggest that such interventions may have potential to influence real-world decision-making. Future research should examine the conditions under which risk communication methods are most likely to influence real-world behaviors and decisions.

In Study 1, we found that many participants’ concerns about COVID did not change from pre- to post-intervention. The reason why this was the case is unclear. We present an exploratory analysis examining some of the factors that may have contributed to one’s willingness to increase their concern post-intervention. We found evidence that participants who identified with a right-leaning political ideology were less likely to increase their concern than left-leaning participants. This suggests that right- and left-leaning individuals may process data differently from one another, and that researchers should consider how to best communicate risk to both groups. Political partisanship is just one factor that could influence a person’s willingness to update their concern about a risk in the face of evidence. Future studies should investigate the factors that influence one’s openness to updating their beliefs.

We would like to note that in the specific example used in this study, there was an optimal estimation of risk (i.e., 40%). However, the translation of that estimate to concern will vary by individual. For example, an extremely ris- averse person or someone with  a pre-existing condition may perceive the risk of 40% as greater than another individual would. Whether one is overestimating or underestimating possible risks depends on many individual factors, as well as the consequences of misestimating. In the specific example we present, we do not think thatparticipants could have been overly concerned. People should be very concerned about a 40% chance of dining with an individual with COVID or a hypothetical illness. However, future work could consider  how different types of data presentations may influence risk perception when the risk is small (such as vaccine side effects).

In Study 1, we found evidence that although participants in the Static Condition reported an increase in perceived risk of contracting COVID, their perceived concern *decreased*. This suggests that participants remembered the risk-related information presented to them in the intervention (i.e., the risk of contracting COVID at a Thanksgiving dinner with 10 people was 40%); however, they were less concerned about the risk. It is unclear why there was a disconnect between their risk perception and concern. Future research could use additional methods—like qualitative free-response analysis - to explain this phenomenon.

Lastly, participants in the Static Condition in Study 1 demonstrated this backfire effect, but this was not observed in the second study. There were many important differences between Studies 1 and 2, namely, Study 1 was conducted in the context of understanding *real-world* data, while Study 2 was conducted in the context of reasoning about *hypothetical* data. Other notable differences include the fact that that Study 1 presented data as a bar chart, while Study 2 presented it as an icon array, and that the Study 1 data analysis only included participants with low-risk perception (i.e., they planned to dine with others during the 2020 Thanksgiving holiday). It is worth investigating the conditions under which these backfire effects occur. We would also like to that in Fig. 6, there is an outlier data point for the Static Condition (decrease in concern by 100 points). We still observed the data backfire effect even after excluding this individual’s data.

### Limitations

While our research showed an advantage for narrative visualizations compared to static visualizations, it is unclear whether this advantage would also be found in other scenarios and contexts. The context used here was one for which risk was (essentially) cumulative. Cumulative risk can be especially challenging to comprehend, in which case, a visualization like the narrative visualization may have been particularly helpful. It is an open research question as to whether narrative visualizations would help illustrate risks of various magnitudes. Narrative visualizations could also be explored as a tool to help people understand both absolute and relative risk (such as a difference between 2% and 4%, which is a relative difference of 100%).

One limitation of our work was our method of measuring trust. Participants were given two Likert-scale items to measure trust in the visualization, measuring the perceived intentions of the visualization designer and the perceived accuracy of the data (I feel like the data are intended to accurately portray the risks of the new disease/I feel like the data do accurately portray the risks of the new disease). While it is not uncommon to use such Likert-scale items in research on trust in research (for a review, see Elhamadi et al., 2023), other researchers argue for more holistic approaches that accurately capture the various dimensions of trust. For example, trust may be assessed with various methods including quantitative surveys from social sciences, trust games from behavioral economics, measuring belief updating, and measuring trust through perceptual methods (Elhamdadi et al., 2022b). Elhamdadi et al. (2023) presented an integrated framework for trust in visualization, offering suggestions on how researchers can best capture the various dimensions of trust. The authors suggest that trust in visualization is contingent upon cognitive and affective factors including visualization clarity, usability, accuracy, aesthetics, and the extent to which the visualization is an accurate representation of the underlying data (benevolence/ethics). While our measures assessed two facets of trust, future research in this area should use a more comprehensive assessment of trust to better understand how narrative visualizations impact the different components of trust. While not ideal, we do find some evidence for reliability and validity of the measures we present, as the two items had high reliability ($\alpha$ = 0.91), and our measure of trust related to understanding, visualization presence, and concern in the directions one would expect.

We found that trust in the visualization, via understanding, mediated the effect of the narrative visualization on posttest concern. However, we found partial mediation and not full mediation. This raises the open question of which other aspects of the narrative visualization contributed to  its effectiveness. Answering this question would be useful for establishing design principles for narrative visualizations. For example, it could be useful to run a version of the study where participants read about how the 40% risk was calculated, but they do not view the accompanying visualizations. It is also possible that time on task influenced subsequent concern and risk perception. The current study did not collect data on timin;, however,  the amount of time interacting with each intervention may have had an influence. Lastly, it is unclear if viewing the narrative visualization actually improved understanding of the data-generating process. Future research should include objective measures of understanding to determine whether actual understanding maps onto perceived understanding.

### Conclusion

The presented work investigated the promise of narrative visualizations for communicating data about cumulative risk. The narrative visualizations implemented in our intervention included icon arrays and accompanying text describing how one’s risk of contracting a disease accumulates as the number of people one interacts with increases. Our results provide promising evidence that narrative visualizations are an effective tool formitigatingbackfire effects by increasing understanding and subsequent trust in data. This work suggests that misinformation correction does not need to rely on emotion-laden content or interventions that affect risk perception through heuristic thinking; indeed, it appears possible that narrative visualizations may be an effective way to improve risk perception using quantitative data.

## Supplementary Information

Supplementary Material 1

## Data Availability

The datasets supporting the conclusions of this article are available in the OSF repository, https://osf.io/k43ev/?view_only=67cc6401b06946889ac499fc19afec2f.
