# Supplementary material for: Narrative visualizations: Depicting accumulating risks and increasing trust in data
Source: Cogn Res Princ Implic. 2025 Feb 21;10:7. doi: 10.1186/s41235-025-00613-w (PMC11845644; doi:10.1186/s41235-025-00613-w)
Supplement: Supplementary file 1 — Supplementary Material 1 [file 41235_2025_613_MOESM1_ESM.docx]

**Supplemental Materials for: Increasing Trust in Data Through Narrative Visualizations**

[Study 1 - Full Anecdote 2](#_3d0kzjmcud14)

[Study 1 - Pre-Intervention Survey Items 3](#_khphzyvln8n9)

[Study 1 - Post-Intervention Survey Items 4](#_e6p6cew585g6)

[Study 1 - Post-Thanksgiving Follow-up Survey 6](#_dpyt12mve5a6)

[Study 2 - Full Anecdote 8](#_qm356b28s42u)

[Study 2 - Pre-Intervention Survey Items 9](#_qx2v1pe23i4f)

[Please answer some questions about what your Thanksgiving plans would have been this year if you were living through the above hypothetical scenario. 9](#_euvx4ycy0q7l)

[Study 2 - Post-Intervention Survey Items 10](#_pcavuubjwczh)

#

# **Study 1 - Full Anecdote**

Please read the story and then continue.

Melissa (37F, Canada)

Nov. 3, 2020

My family is not one to take risks. When the virus started taking off in late March, we were very careful. Both of our kids’ schools closed as a precaution, and we took extra steps to protect ourselves at home. We bought more groceries to cut down on trips, we stopped eating out, and we (grudgingly) cancelled the spring cabin retreat we had planned with friends. In April, cases continued to mount. Both my husband and I began working from home, and school resumed online for the kids. It was hard staying indoors all the time, but we managed to find ways to entertain ourselves (the kids convinced us to buy them a Nintendo Switch, which admittedly did keep them occupied). Summer arrived, and the warm weather let us get outside more (hiking and bike rides, mostly), which helped a lot especially since school was out for the kids.

When the weather started to cool off in the Fall, it was easier to deal with: cases were declining and things began to re-open (much to the kids’ delight, we resumed our tradition of Friday-night pizza). The pandemic wasn’t over, but at least it felt like things were starting to get back to normal. When it came time for Canadian Thanksgiving, we decided to drive to my parents’ house for turkey dinner together with my brother and his wife. It seemed safe enough, since we would only be 8 people and we’re all family (plus I knew my parents were missing their grandchildren dearly). But that seemingly harmless decision is something I will probably regret for the rest of my life.

Two days after Thanksgiving I felt incredibly tired -so much so that I called in sick at work. My husband slept on the couch while I tried to isolate myself in the bedroom. By evening I developed a fever of 104 along with a burning cough. I couldn’t sleep, and around 2 am I began to panic. It felt like I had cotton balls in my throat and nostrils, it was getting harder and harder to breathe. I woke my husband and he drove me to the hospital. After a couple of terrifying hours, the nurses were able to get me stabilized (though still on oxygen), but the relief was short-lived. We got a call from my mom later that day: an ambulance had just driven my dad to the ICU.

My husband and kids all tested positive, but they didn’t show any symptoms beyond sniffles. On the other hand, I’m at home now after two weeks in the hospital, but I am nowhere near recovered. I still need a thin tube with a portable oxygen tank to breathe comfortably, and if I walk for more than a couple minutes at a time I feel exhausted. On top of that, my resting heartbeat is abnormally high (the doctors told me they’ve seen this in a few other COVID patients). But I’m the lucky one. My dad is still intubated and spends most of the day sedated because his body’s breathing won’t sync up with the machine. The doctor on call told us to prepare for the worst but still, hold onto our hope.

I’m so scared, and words can’t describe the guilt I feel. I know I’m just one person, but if you take anything away from this story please STAY HOME. The pandemic has been hard for everyone, and we all miss our families. But please, don’t risk something like this happening to you or your family. Stay home not just to protect yourself, but to protect your loved ones too.

#

# **Study 1 - Pre-Intervention Survey Items**

1. Who will you celebrate Thanksgiving with (in person)? (Check all that apply)
   1. Alone/with people who live with you
   2. Extended family
   3. Friends/neighbors
   4. Strangers
2. Where will you celebrate Thanksgiving?
   1. At your home
   2. At someone else’s home
   3. Community/public event
3. How concerned are you about getting COVID-19 at Thanksgiving?
   1. 0 (not concerned at all) - 100 (extremely concerned)
4. How concerned are you that someone in your family will get COVID-19 at Thanksgiving?
   1. 0 (not concerned at all) - 100 (extremely concerned)
5. Are you or anyone at your Thanksgiving dinner table going to be traveling?
   1. No
   2. Yes, by car
   3. Yes, by public transportation
   4. Yes, by plane
6. How many people beyond your household will be at Thanksgiving dinner (select 21 if there will be more than 20 people)?
   1. Slider scale of 0 - 21
7. Has Coronavirus changed your plans this year?
   1. No
   2. Yes (I am spending Thanksgiving with fewer people than planned)
   3. Yes (I am spending Thanksgiving with more people than planned)
8. What is the risk that at least one person at a Thanksgiving table with 10 people has COVID?
   1. Slider scale of 0% to 100%

#

# **Study 1 - Post-Intervention Survey Items**

**Anecdote Condition**

1. After reading the story, what do you think is the risk that at least one person at a Thanksgiving table with 10 people has COVID-19?
   1. Slider scale of 0% to 100%
2. After reading the story, how concerned are you about getting COVID-19 at Thanksgiving?
   1. 0 (not concerned at all) - 100 (extremely concerned)
3. After reading the story, how concerned are you that someone in your family will get COVID-19 at Thanksgiving?
   1. 0 (not concerned at all) - 100 (extremely concerned)
4. After reading the story, how do you feel about your current plans?
   1. 0 (not concerned at all) - 100 (extremely concerned)

**Static/Data Walkthrough Conditions**

1. Based on the graphs, what do you think is the risk that at least one person at a Thanksgiving table with 10 people has COVID-19?
   1. Slider scale of 0% to 100%
2. After viewing these data, how concerned are you about getting COVID-19 at Thanksgiving?
   1. 0 (not concerned at all) - 100 (extremely concerned)
3. After viewing these data, how concerned are you that someone in your family will get COVID-19 at Thanksgiving?
   1. 0 (not concerned at all) - 100 (extremely concerned)
4. After viewing these data, how do you feel about your current plans?
   1. 0 (not concerned at all) - 100 (extremely concerned)

**Static + Anecdote Condition**

1. After viewing these data and reading the story, what do you think is the risk that at least one person at a Thanksgiving table with 10 people has COVID-19?
   1. Slider scale of 0% to 100%
2. After viewing these data and reading the story, how concerned are you about getting COVID-19 at Thanksgiving?
   1. 0 (not concerned at all) - 100 (extremely concerned)
3. After viewing these data and reading the story, how concerned are you that someone in your family will get COVID-19 at Thanksgiving?
   1. 0 (not concerned at all) - 100 (extremely concerned)
4. After viewing these data and reading the story, how do you feel about your current plans?
   1. 0 (not concerned at all) - 100 (extremely concerned)

**All Conditions**

1. What are your plans for the December holidays?
   1. More likely to interact with fewer people than I normally would
   2. More likely to interact with more people than I normally would
   3. More likely to interact with as many people than I normally would
   4. Undecided
2. How confident are you that your December holiday plans strike a good balance between life-as-normal and being safe?
   1. 0 (not confident at all) - 100 (extremely confident)
3. What is your age?
4. What is your zip code?
5. What is your gender?
6. What age did you begin learning English?
7. What language do you use most at home?
8. What is your highest level of education completed?
9. Do you personally know someone who has COVID?
10. Subjective Numeracy Scale (SNS-3; McNaughton et al., 2015)
11. Please list one to three of your primary sources of information (e.g., “New York Times”, “Fox News”, etc.)
12. What is your political affiliation?

#

# **Study 1 - Post-Thanksgiving Follow-up Survey**

1. Do you plan to attend or host/have attended or hosted any in-person gatherings with people outside of your household for the December/January holidays?
   1. Yes
   2. No
   3. Unsure
2. How many in-person gatherings with people outside of your household do you expect to attend or host/have attended in the or hosted?
   1. Slider scale of 0-10
3. What is the total number of people you expect to attend the largest event you plan to attend/have attended? (Enter a number excluding yourself)
4. Are you or anyone at your holiday events dinner table going to be traveling/have traveled?
   1. No
   2. Yes, by car
   3. Yes, by public ground transportation
   4. Yes, by plane
5. How has Coronavirus changed your plans this year?
   1. No change
   2. I am spending the holidays with fewer people than planned
   3. I am spending the holidays with more people than planned
6. Do you plan to have an indoor meal with people outside of your household within the next 4 weeks?
   1. Yes
   2. No
   3. Not Sure
7. Do you plan to attend an indoor religious service within the next 4 weeks?
   1. Yes
   2. No
   3. Not Sure
8. Based on the previous Survey of Holiday Plans, what is the risk that at least one person at a Thanksgiving table with 10 people might have COVID-19?
   1. Slider scale of 0% to 100%
9. In the previous *Survey of Holiday Plans (< 5 min)*, which information below did you receive?
   1. Melissa’s experience with COVID-19
   2. Melissa’s experience with COVID-19 accompanied by a bar graph showing your risk of running into a COVID-19 positive person at a 10-person Thanksgiving dinner
   3. A bar graph showing your risk of running into a COVID-19 positive person at a 10-person Thanksgiving dinner
   4. A set of illustrations showing you how there is such risk of running into a COVID-19 positive person at a 10-person Thanksgiving dinner
   5. I don’t remember
10. Before participating in the previous study, had you ever seen a story or data graph like the one in the study?
    1. Yes
    2. No
    3. I don’t remember
11. How many COVID-19 related studies have you participated in?
    1. Slider scale of 0-100
12. Did you make any last-minute changes to your Thanksgiving plans?
    1. Yes
    2. No
13. What last-minute changes did you make to your Thanksgiving plans?
    1. Free response
14. Did your change of plans increase or decrease the number of people you interacted with during Thanksgiving?
    1. It increased the number of people I interacted with during Thanksgiving
    2. It decreased the number of people I interacted with during Thanksgiving
    3. It neither increased or decreased the number of people I interacted with during Thanksgiving
15. How many people (besides yourself) from each of the following categories did you celebrate Thanksgiving in-person with? (Please enter a number for each of the categories below & enter '0' if you had no interaction with people from that category)
    1. People who live with you (free response)
    2. Extended family who live outside your household (free response)
    3. Friends/neighbors who live outside your household (free response)
    4. Strangers
16. Where did you celebrate Thanksgiving?
    1. At your home
    2. At someone else’s home
    3. Restaurant/Public/Community event
17. How concerned are you about getting COVID-19 / Coronavirus via social gatherings in December?
    1. 0 (not concerned at all) - 100 (extremely concerned)
18. How concerned are you that someone in your family will get COVID-19 via social gatherings in December?
    1. 0 (not concerned at all) - 100 (extremely concerned)
19. How risky do you think it is to interact with people outside of your household for a holiday meal?
    1. 0 (not at all risky) - 100 (extremely risky)
20. How risky do you think it is to interact with people outside of your household to attend an indoor religious service?
    1. 0 (not at all risky) - 100 (extremely risky)
21. In the last two weeks, how often have you sought out Covid-related scientific information?
    1. 0 (Never) - 100 (More than once a day)
22. In the last two weeks, how often have you sought out Covid-related scientific information compared to prior weeks?
    1. 0 (Less) - 10 (More)

# **Study 2 - Full Anecdote**

Please read the story and then continue.

Melissa (37F, Michigan)

My family is not one to take risks. When the virus started taking off, we were very careful. Both of our kids’ schools closed as a precaution, and we took extra steps to protect ourselves at home. We bought more groceries to cut down on trips, we stopped eating out, and we (grudgingly) canceled the spring cabin retreat we had planned with friends. As cases continued to mount, both my husband and I began working from home, and school resumed online for the kids. It was hard staying indoors all the time, but we managed to find ways to entertain ourselves (the kids convinced us to buy them a Nintendo Switch, which admittedly did keep them occupied). Summer arrived, and the warm weather let us get outside more (hiking and bike rides, mostly), which helped a lot especially since school was out for the kids.

When the weather started to cool off in the Fall, it was easier to deal with: cases were declining and things began to re-open (much to the kids’ delight, we resumed our tradition of Friday-night pizza). The pandemic wasn’t over, but at least it felt like things were starting to get back to normal. When it came time for Thanksgiving, we decided to drive to my parents’ house for turkey dinner together with my brother and his wife / children. It seemed safe enough, since we would only be 10 people and we’re all family (plus I knew my parents were missing their grandchildren dearly). But that seemingly harmless decision is something I will probably regret for the rest of my life.

Two days after Thanksgiving I felt incredibly tired -so much so that I called in sick at work. My husband slept on the couch while I tried to isolate myself in the bedroom. By evening I developed a fever of 104 along with a burning cough. I couldn’t sleep, and around 2 am I began to panic. It felt like I had cotton balls in my throat and nostrils, it was getting harder and harder to breathe. I woke my husband and he drove me to the hospital. After a couple of terrifying hours, the nurses were able to get me stabilized (though still on oxygen), but the relief was short-lived. We got a call from my mom later that day: an ambulance had just driven my dad to the ICU.

My husband and kids all tested positive, but they didn’t show any symptoms beyond sniffles. On the other hand, I’m at home now after two weeks in the hospital, but I am nowhere near recovered. I still need a thin tube with a portable oxygen tank to breathe comfortably, and if I walk for more than a couple minutes at a time I feel exhausted. On top of that, my resting heartbeat is abnormally high (the doctors told me they’ve seen this in a few other patients). But I’m the lucky one. My dad is still intubated and spends most of the day sedated because his body’s breathing won’t sync up with the machine. The doctor on call told us to prepare for the worst but still, hold onto our hope.

I’m so scared, and words can’t describe the guilt I feel. I know I’m just one person, but if you take anything away from this story please STAY HOME. The pandemic has been hard for everyone, and we all miss our families, but the risks can be much higher than we realize. I found out afterward that (according to the CDC) even though only about 4 out of 100 people are infected, there was about a 40% chance that someone at our dinner of 10 was infected. So please don’t risk something like this happening to you or your family. Stay home not just to protect yourself, but to protect your loved ones too.

# **Study 2 - Pre-Intervention Survey Items**

Imagine the following scenario: a **new respiratory disease** emerged in your community. This new disease is **highly contagious**, and there is **no vaccine available** yet. The Fall & Winter holiday season is approaching and public health officials recommend that people do not gather during the holidays, but your local government has not placed any restrictions on gathering.

### Please answer some questions about what your Thanksgiving plans would have been this year **if you were living through the above hypothetical scenario.**

1. In this scenario, who would you have Thanksgiving dinner with (in person)? (check all that apply)
   1. Alone/with people who live with you
   2. Extended family
   3. Friends/neighbors
   4. Strangers
2. In this scenario, where would you celebrate Thanksgiving?
   1. At your home
   2. At someone else’s home
   3. Community/public event
3. How concerned are you about contracting the disease at Thanksgiving?
   1. 0 (not concerned at all) - 100 (extremely concerned)
4. How concerned are you that someone else at your Thanksgiving dinner will contract the disease?
   1. 0 (not concerned at all) - 100 (extremely concerned)
5. Would a disease like the one in this scenario change your Thanksgiving plans compared to a typical year?
   1. No change
   2. Yes (I would spend Thanksgiving with fewer people than planned)
   3. Yes (I would spend Thanksgiving with more people than planned)
6. If 4% of the population has the disease, what is the risk that at least one person at a Thanksgiving table with 10 people has the disease?
   1. Slider scale from 0% - 100%

# **Study 2 - Post-Intervention Survey Items**

***Anecdote Condition***

1. According to the data at the end of the story, what is the approximate risk that you will be exposed to the disease if 4 out of 100 people in your community are infected and you have dinner with 10 people?
   1. 40%
   2. 4%
   3. 10%
   4. 14%
   5. 90%
2. After reading the story, how concerned are you about contracting the disease at Thanksgiving?
   1. Slider scale from 0 (*not concerned at all*) to 100 (*extremely concerned*)
3. After reading the story, how concerned are you that someone else at your Thanksgiving dinner will contract the disease?
   1. Slider scale from 0 (*not concerned at all*) to 100 (*extremely concerned*)
4. How do you feel about the Thanksgiving plans you reported before reading the story?
   1. Slider scale from 0 (*not concerned at all*) to 100 (*extremely concerned*)
5. After reading the story, I understand why a Thanksgiving dinner with 10 people has about a 40% chance of exposure to the disease.
   1. Slider scale from 0 (*strongly disagree*) to 100 (*strongly agree*)
6. I feel like the story is **intended** to accurately portray the risks of the new disease.
   1. Slider scale from 0 (*strongly disagree*) to 100 (*strongly agree*)
7. I feel like the story **does** accurately portray the risks of the new disease.
   1. Slider scale from 0 (*strongly disagree*) to 100 (*strongly agree*)

***All Other Conditions***

1. According to the data at the end of the story, what is the approximate risk that you will be exposed to the disease if 4 out of 100 people in your community are infected and you have dinner with 10 people?
   1. 40%
   2. 4%
   3. 10%
   4. 14%
   5. 90%
2. After viewing these data, how concerned are you about contracting the disease at Thanksgiving?
   1. Slider scale from 0 (*not concerned at all*) to 100 (*extremely concerned*)
3. After viewing these data, how concerned are you that someone else at your Thanksgiving dinner will contract the disease?
   1. Slider scale from 0 (*not concerned at all*) to 100 (*extremely concerned*)
4. How do you feel about the Thanksgiving plans you reported before viewing the data?
   1. Slider scale from 0 (*not concerned at all*) to 100 (*extremely concerned*)
5. After viewing the data, I understand why a Thanksgiving dinner with 10 people has about a 40% chance of exposure to the disease.
   1. Slider scale from 0 (*strongly disagree*) to 100 (*strongly agree*)
6. I feel like the data are **intended** to accurately portray the risks of the new disease.
   1. Slider scale from 0 (*strongly disagree*) to 100 (*strongly agree*)
7. I feel like the data **do** accurately portray the risks of the new disease.
   1. Slider scale from 0 (*strongly disagree*) to 100 (*strongly agree*)

**All Conditions**

1. In the hypothetical scenario you read at the beginning of the study, was a vaccine available for the disease?
   1. Yes, one vaccine was available.
   2. Yes, multiple vaccines were available.
   3. No, vaccines were not available.
2. What is your age? (Please enter a number)
3. What is your zip code?
4. What is your gender?
5. What age did you begin learning English? (Please answer in the form of a number: if you are a native speaker, please write "0")
6. What language do you use most at home?
7. What is your highest level of education completed? (5 - elementary school; 8 - last year in middle school; 12 - senior year in high school; 16 - senior year in college)
8. Do you personally know someone who has been hospitalized due to COVID-19?
9. Are you currently vaccinated against COVID-19? (select all that apply)
   1. Yes (by choice)
   2. Yes (mandated by job or government)
   3. No (by choice)
   4. No (unavailable or medical condition)
   5. Prefer not to say
10. Subjective Numeracy Scale (SNS-3; McNaughton et al., 2015)
11. Please list one to three of your primary sources of information (e.g., “New York Times”, “Fox News”, etc.)
12. What is your political affiliation?
